# Genome-Wide Association Study Reveals Genomic Regions Associated With Molybdenum Accumulation in Wheat Grains

**DOI:** 10.3389/fpls.2022.854966

**Published:** 2022-03-02

**Authors:** Xiaojie Jin, Zhaojun Zou, Zhengqing Wu, Congcong Liu, Songxian Yan, Yanchun Peng, Zhensheng Lei, Zhengfu Zhou

**Affiliations:** ^1^Hubei Key Laboratory of Food Crop Germplasm and Genetic Improvement, Institute of Food Crops, Hubei Academy of Agricultural Sciences, Wuhan, China; ^2^Henan Institute of Crop Molecular Breeding, Henan Academy of Agricultural Sciences, Zhengzhou, China; ^3^College of Plant Science and Technology, Huazhong Agricultural University, Wuhan, China; ^4^Department of Resources and Environment, Moutai Institute, Renhuai, China

**Keywords:** common wheat, molybdenum, GWAS, SNP, candidate genes

## Abstract

Molybdenum (Mo) is an essential micronutrient for almost all organisms. Wheat, a major staple crop worldwide, is one of the main dietary sources of Mo. However, the genetic basis for the variation of Mo content in wheat grains remains largely unknown. Here, a genome-wide association study (GWAS) was performed on the Mo concentration in the grains of 207 wheat accessions to dissect the genetic basis of Mo accumulation in wheat grains. As a result, 77 SNPs were found to be significantly associated with Mo concentration in wheat grains, among which 52 were detected in at least two sets of data and distributed on chromosome 2A, 7B, and 7D. Moreover, 48 out of the 52 common SNPs were distributed in the 726,761,412–728,132,521 bp genomic region of chromosome 2A. Three putative candidate genes, including molybdate transporter 1;2 (TraesCS2A02G496200), molybdate transporter 1;1 (TraesCS2A02G496700), and molybdopterin biosynthesis protein CNX1 (TraesCS2A02G497200), were identified in this region. These findings provide new insights into the genetic basis for Mo accumulation in wheat grains and important information for further functional characterization and breeding to improve wheat grain quality.

## Introduction

As a critical component of enzymes that catalyze key reactions in nitrogen, carbon, and sulfur metabolism, molybdenum (Mo) is an essential micronutrient required for the growth and development of plants and animals (Arnon and Stout, 1939; Turnlund, 2002; Mendel and Bittner, 2006). Higher plants and animals absorb or take in Mo as oxyanion molybdate, which becomes biologically active by binding to pterin to form Mo cofactor (Moco; Mendel and Bittner, 2006; Mendel, 2013). Thereafter, Moco participates in the synthesis of molybdate-dependent enzymes (Molybdoenzymes), including nitrate reductase, sulfite oxidase, xanthine oxidoreductase/dehydrogenase, aldehyde oxidase, and mitochondrial amidoxime reducing component (Hille et al., 2011; Bittner, 2014). To date, a number of molybdate transporters have been identified in plants, including AtMOT1;1 and AtMOT1;2 in *Arabidopsis thaliana* (Tomatsu et al., 2007; Baxter et al., 2008; Gasber et al., 2011), OsMOT1;1 and OsMOT1;2 in *Oryza sativa* (Yang et al., 2018; Huang et al., 2019; Ishikawa et al., 2021), FaMOT1 in strawberry (Liu et al., 2020b), LjMOT1 in *Lotus japonicus* (Gao et al., 2016; Duan et al., 2017), and MtMOT1.2 and MtMOT1.3 in *Medicago truncatula* (Tejada-Jimenez et al., 2017; Gil-Diez et al., 2019).

Mo deficiency frequently occurs in plants when grown in acidic soils with low Mo bioavailability. Mo-deficient plants generally show overall impaired plant growth and decrease in productivity (Kaiser et al., 2005). Moreover, Mo content in seeds may positively affect seedling vigor in acidic soils (Modi and Cairns, 1994; Modi, 2002; Norton et al., 2014). In humans, Moco deficiency (MoCD) will lead to metabolic defects in molybdoenzymes, giving rise to the accumulation of sulfite, taurine, S-sulfocysteine, and thiosulfate. MoCD is a rare genetic disease, which causes neurological disorders and ultimately early death (Johnson and Duran, 2001; Atwal and Scaglia, 2016). In addition, a molybdenum compound, tetrathiomolybdate, has been clinically used to treat the Wilson’s disease, a genetic disorder of copper metabolism (Brewer et al., 2009).

Wheat (*Triticum aestivum* L.) is a major staple food crop worldwide, providing ~25% of calories and nutrients in human diet (Zhou et al., 2021b). Food products of wheat grains are one of the primary sources of dietary Mo for adults (Novotny, 2011; Novotny and Peterson, 2018). However, Mo deficiency is a widespread problem of micronutrient deficiency in agriculture. In Australia, Mo deficiency generally occurs in large areas of cropland with acidic soils and has been identified as the second most common micronutrient deficiency after Zinc (Holloway et al., 2008). In China, more than 44.6 million ha of arable land is Mo-deficient (Zou et al., 2008; Nie et al., 2014). Wheat plants grown in these Mo-deficient soils exhibit severe Mo deficiency symptoms such as pale green leaves, chlorosis of seedlings, and lower yields (Yu et al., 2010). Therefore, dissecting the genetic basis and the molecular mechanism for Mo accumulation in wheat grains will greatly help to improve wheat yield and quality.

In previous studies, to understand the genetic mechanism for Mo accumulation in plants, a number of loci and genes related to Mo accumulation have been identified in several species using the genome-wide association study (GWAS) strategy. For example, molybdate transporter 1 (MOT1), which controls the natural variation of Mo concentration in *A. thaliana* leaves, was identified by GWA mapping (Shen et al., 2012; Forsberg et al., 2015). In rice, Norton et al. (2014) found a significant locus for Mo accumulation in grains on chromosome 8, which contains the *MOT1* orthologue. Yang et al. (2018) analyzed the Mo concentration in rice grains by GWAS and demonstrated that variations of Mo concentration in rice grains can be attributed to variable expression of *OsMOT1;1*. Furthermore, Cobb et al. (2021) also found strong genetic signals for Mo concentration in the shoot in the genomic region of the *OsMOT1;1* gene on rice chromosome 8 by GWAS analysis. In wheat, GWAS has been employed to analyze the concentrations of several minerals in the grains, such as zinc (Alomari et al., 2018; Velu et al., 2018; Cu et al., 2020; Zhou et al., 2020), iron (Alomari et al., 2019; Cu et al., 2020), and calcium (Alomari et al., 2017). However, no GWAS has been carried out on grain Mo concentration (GMoC) in wheat to our knowledge.

In this study, by using a panel of 207 wheat accessions, we aimed to: (i) explore the genetic variation of GMoC in wheat; (ii) identify the genomic regions associated with wheat GMoC using the GWAS approach; and (iii) identify the candidate genes for the variations of wheat GMoC. The results of the present study may facilitate the development of wheat varieties with improved nutritional quality.

## Materials and Methods

### Plant Materials and Growth Conditions

A total of 207 common wheat accessions were used in this study, including both elite cultivars and landraces mainly from China and seven other nations (Supplementary Table 1). These accessions were grown in Yuanyang (35°5′N, 113°97′E), Henan province, a main wheat growing area of China during the 2018–2019 and 2019–2020 cropping seasons in a randomized complete block design. Each genotype was planted in two rows of 2 m length with a 0.3 m row spacing. Field management was conducted based on standard agronomic practices.

### Determination of Grain Mo Concentration

At maturity, wheat grains were harvested, dried at 55°C for 24 h, and milled to fine powders. Then, the milled samples were dried at 55°C for another 24 h. After that, 200 mg of dried powder for each sample was digested in 8.0 ml HNO_3_ in a microwave reactor with a gradient of temperature from 120°C to 180°C for 30 min. After dilution in deionized distilled water, the Mo concentration was measured by inductively coupled plasma mass spectrometry (ICP-MS, NexION 1,000, Perkin Elmer, United States).

### Statistical Analysis

The best linear unbiased predictor (BLUP) of Mo concentration for each accession across the 2 years was calculated using the R package lme4 (Bates et al., 2015). The broad-sense heritability (*H*^2^) was calculated using the equation $H^{2}=\sigma_{g}^{2}/\left(\sigma_{g}^{2}+\sigma_{e}^{2}/e\right)$ , where σ^2^_g_ is the variance of genotype, σ^2^_e_ is the variance of environment, and e is the number of years (*e* = 2 in this study). Pearson’s correlation coefficient (r) for the grain Mo trait across the 2 years was calculated in R version 4.1.1.

### SNP Genotyping and GWAS

All wheat accessions were genotyped using the Wheat 660 K SNP array. To avoid spurious SNPs, the SNPs with minor allele frequency (MAF) < 0.05 and missing data >10% were removed. After filtering, a total of 224,706 SNPs were used for GWAS. The population structure and kinship matrix of the association panel were calculated as described in a previous study (Liu et al., 2020a).

GWAS for grain Mo concentration was performed by a mixed-model approach with the FaST-LMM (factored spectrally transformed linear mixed models) program (Lippert et al., 2011). The effective number of SNPs (*n* = 37,350 in this study) was calculated with the GEC software (Li et al., 2012). Accordingly, the Bonferroni value of p threshold of 2.68E-05 (*p* = 1/*n; n* is the effective number of SNPs) was used to determine significant SNPs. R package CMplot^1^ was used to visualize the Manhattan and quantile-quantile (QQ) plots.

### Candidate Gene Identification

To explore the candidate genes responsible for GMoC, genes in the genomic region of 500 Kb upstream and downstream of each significant SNP consistently identified in two or more sets of data were screened based on published slow LD decay in common wheat (Sun et al., 2017; Zhou et al., 2021a). The potential candidate genes were selected based on their annotation information of IWGSC RefSeq v1.1 and functions of homologous genes in *Arabidopsis* and *Oryza sativa*. The phylogenetic tree was constructed by using the Neighbour-Joining (NJ) method in MEGA 11 with a bootstrap of 1,000 replicates (Tamura et al., 2021).

### Gene Expression Level Analysis

As described in a previous study (Liu et al., 2020a), the grains at 20 days after pollination of each wheat accession were collected for RNA-sequencing. The expression levels (FPKM) of the potential candidate genes were extracted for gene expression level analysis. Moreover, the expression levels of these candidate genes in different tissues were downloaded from the Wheat Expression Brower,^2^ which was powered by expVIP platform (Borrill et al., 2016; Ramirez-Gonzalez et al., 2018). Heatmap of the expression levels of the candidate genes was visualized by R package pheatmap.

## Results

### Phenotype Variation of Grain Mo Concentration in Wheat

**Figure 1:**
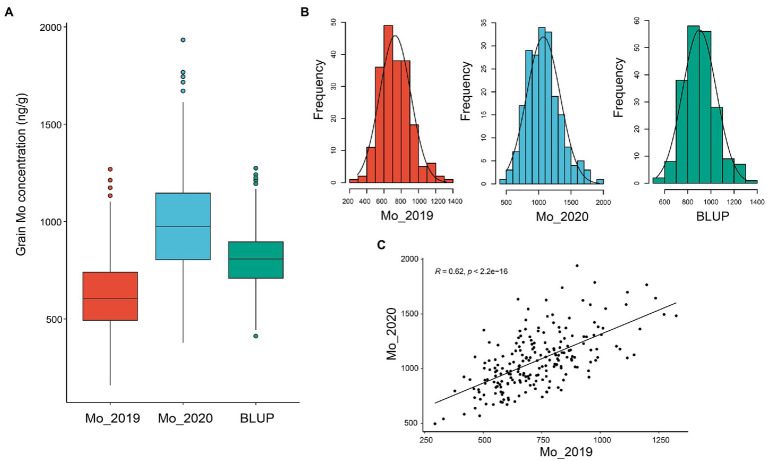
Grain Mo concentration of 207 wheat accessions in 2019 and 2020, and BLUP values. **(A)** Boxplots of grain Mo concentrations for two single years and BLUP values. **(B)** Phenotype distribution of grain Mo concentration (ng/g) in the single season (2019 and 2020) and BLUP value. **(C)** Pearson’s correlation between the two years.

Two hundred and seven wheat accessions, including elite cultivars and landraces from eight nations representing abundant genetic diversity, were grown at Yuanyang, China in the year of 2019 and 2020 to evaluate the Mo concentration in mature grains (Supplementary Table 1). As a result, continuous and extensive variations in grain Mo concentration were observed among different accessions in the 2 years. As shown in Figure 1A, the GMoC in wheat ranged from 496.00 to 1941.58 ng/g, with an average of 1072.67 ng/g and a coefficient of variation (CV) of 24.05% in 2020 (Table 1 and Figure 1A). In the year of 2019, the GMoC ranged from 293.36 to 1324.41 ng/g, with an average of 729.66 ng/g and a CV of 24.67% (Table 1 and Figure 1A). In addition, the BLUP analysis revealed that the average GMoC was 901.16 ng/g and ranged from 528.13 to 1329.19 ng/g across the 2 years. GMoC in 2 years and the BLUP value all showed continuous variations and approximately normal distributions (Figure 1B; Supplementary Figure 1). In addition, the broad-sense heritability (*H*^2^) of GMoC across the 2 years was 0.497, and the Pearson’s correlation coefficient was 0.62 (*p* < 0.001; Figure 1C).

**Table 1:** Summary of grain Mo concentration in 207 wheat accessions.

Environments	Mean (ng/g)	SD	Range (ng/g)	CV (%)	Skewness	Kurtosis
2019	729.66	179.98	293.36–1324.41	24.67	0.57	0.45
2020	1072.67	258.00	496.00–1941.58	24.05	0.59	0.37
BLUP	901.16	145.75	528.13–1329.19	16.17	0.46	0.28

SD, standard deviation; CV, coefficient of variation.

### Genome-Wide Association Study of GMoC

**Figure 2:**
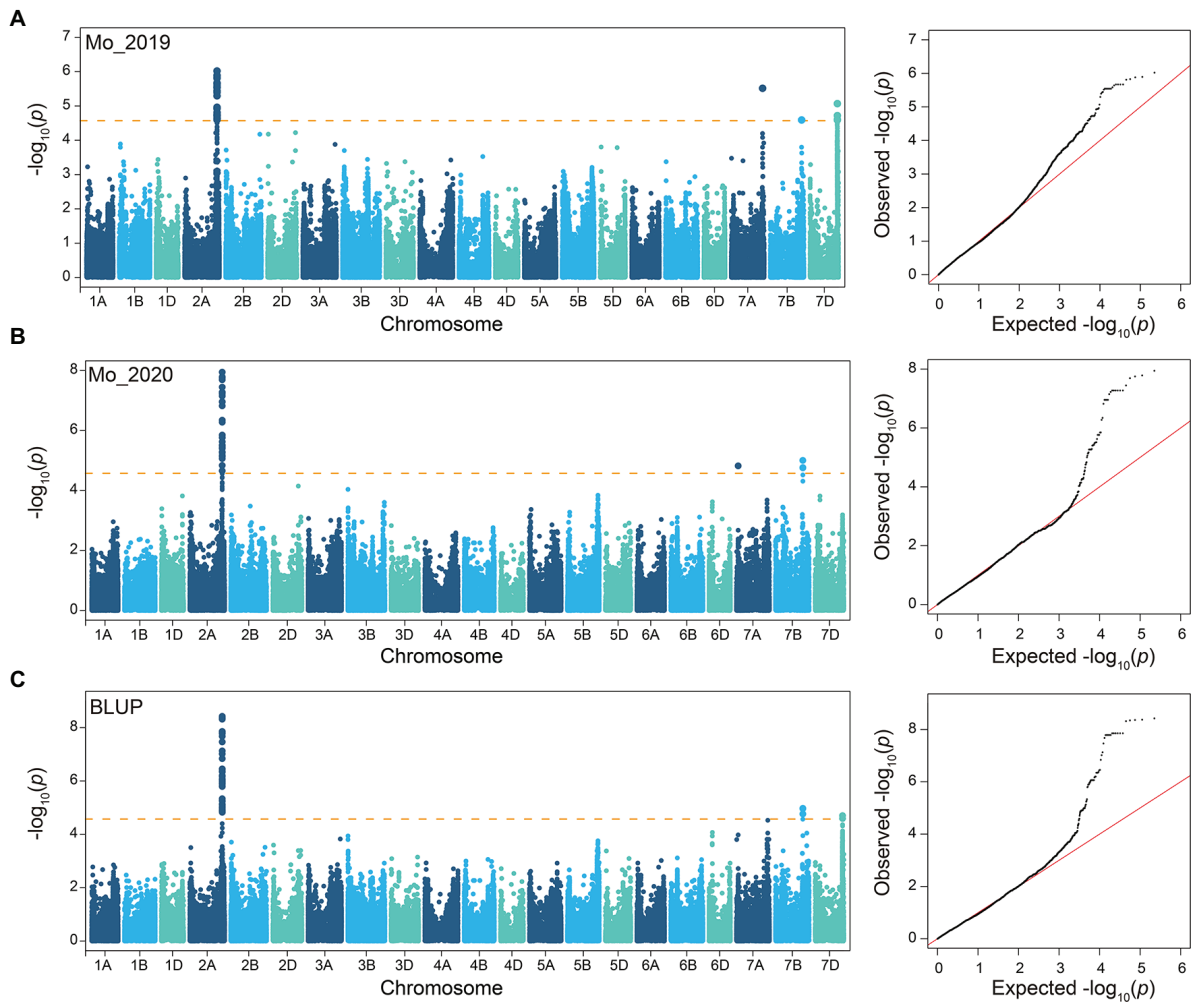
Manhattan plots and QQ plots based on GWAS of GMoC by FaST-LMM model. **(A)** 2019, **(B)** 2020, and **(C)** BLUP values.

**Figure 3:**
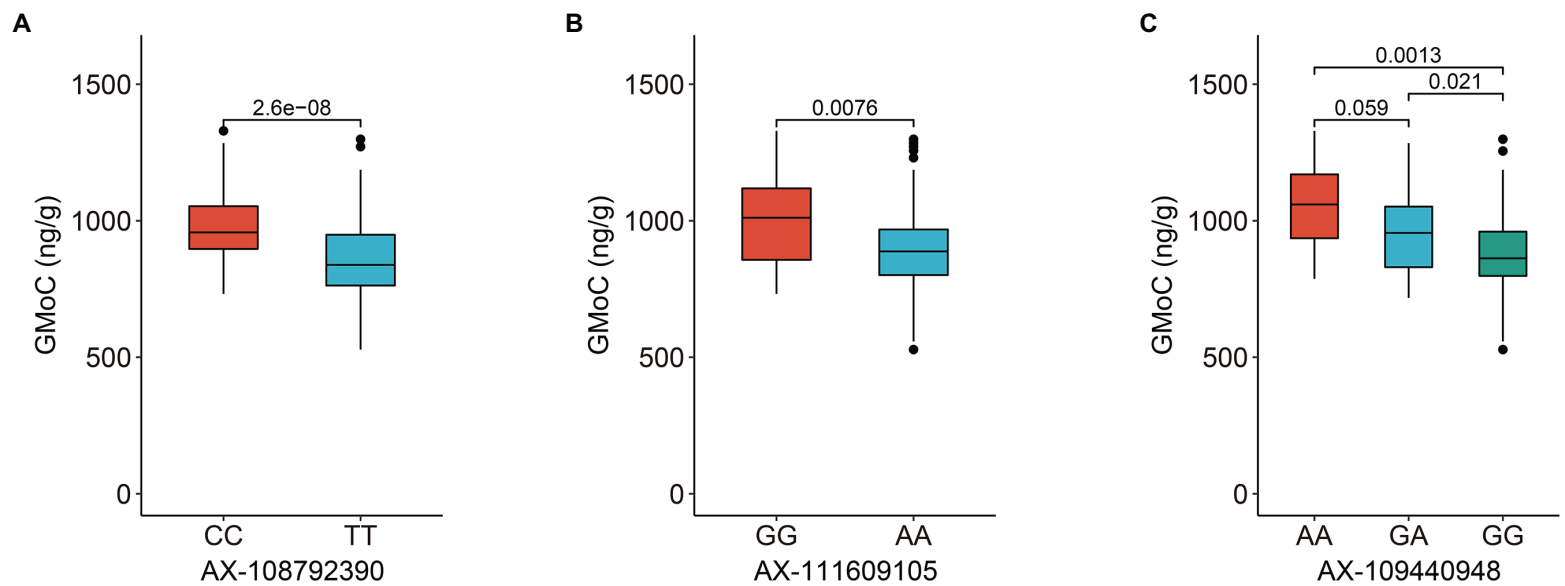
Phenotypic differences in grain Mo concentration of wheat accessions carrying different genotypes of three most significant SNPs on chromosome 2A, 7B, and 7D, respectively. **(A)** AX-108792390 at the position of Chr2A: 727,180,360 bp. **(B)** AX-111609105 at the position of Chr 7B: 611,944,380 bp. **(C)** AX-109440948 at the position of Chr 7D: 611,243,842 bp.

The GMoC data of 207 wheat accessions in 2019 (E1), 2020 (E2), and their BLUP values (B) were employed for GWAS with the 224,706 SNPs using the FaST-LMM program. The Bonferroni value of *p* threshold of 2.68E-05 (−log_10_ (*p*) = 4.57) was used to determine significant SNPs. A total of 45, 51 and 71 SNPs were determined to be significantly associated with GMoC in E1, E2, and BLUP, respectively (Figure 2; Supplementary Table 2). Interestingly, 39, 47, and 66 SNPs associated with GMoC in three analyses (E1, E2, and BLUP) were located in the region of 726,761,412–728,409,806 bp on chromosome 2A, which could explain 7.23–15.94% of the phenotypic variation (PVE). The remaining significantly associated SNPs were located on chromosome 7A, 7B, and 7D, explaining 4.46–13.88% of the phenotypic variation. In BLUP dataset, AX-108792390, AX-111609105, and AX-109440948 were the most significant SNPs on chromosome 2A, 7B, and 7D, respectively. The GMoC was significantly different among wheat accessions carrying different homozygous genotypes of these SNPs (Figure 3; Supplementary Table 3).

The significant SNPs detected in at least two analyses were defined as common SNPs and used for further exploration of candidate genes. Finally, 52 common SNPs were screened, which were distributed on the chromosome of 2A (48 SNPs), 7B (two SNPs), and 7D (two SNPs) with PVE of 5.76–15.94% (Table 2). The significant SNPs detected in the three sets of data were all located on chromosome 2A. The two common SNPs (AX-111609105 and AX-111031595) on chromosome 7B were detected in E2 and B, while the two common SNPs (AX-109440948 and AX-94482751) on chromosome 7D were identified in E1 and B. The GMoC was significantly different among wheat accessions carrying different genotypes at the 52 SNPs (Figure 3; Supplementary Figure 2).

**Table 2:** Summary of common SNPs significantly associated with GMoC by GWAS.

SNP	Chr.	Pos. (bp)	–log_10_( *p*)	PVE (%)	Environment
AX-111651652	2A	726,761,412	4.83, 5.27	10.47, 11.91	E2, B
AX-108892693	2A	726,762,422	4.66, 5.13	10.68, 12.04	E2, B
AX-109972911	2A	726,763,641	4.94–6.20	8.88–11.44	E1, E2, B
AX-109532923	2A	726,763,898	4.94–6.20	8.88–11.44	E1, E2, B
AX-109449153	2A	726,764,021	4.92–6.06	8.82–11.48	E1, E2, B
AX-111515528	2A	726,770,069	4.93–7.02	8.02–12.34	E1, E2, B
AX-109323179	2A	726,771,093	5.43–8.37	8.97–13.49	E1, E2, B
AX-110967398	2A	726,772,232	5.54–7.85	8.84–13.19	E1, E2, B
AX-111698397	2A	726,775,869	5.54–7.85	8.84–13.19	E1, E2, B
AX-111603918	2A	726,776,128	5.83–8.32	10.34–15.94	E1, E2, B
AX-108793615	2A	726,865,730	5.45–7.79	8.87–13.19	E1, E2, B
AX-108777425	2A	726,971,223	5.54–7.85	8.84–13.19	E1, E2, B
AX-109930704	2A	726,988,053	5.81–8.36	10.05–15.14	E1, E2, B
AX-110554284	2A	726,994,887	5.40–7.68	9.34–13.45	E1, E2, B
AX-110492897	2A	727,004,090	5.54–7.85	8.84–13.19	E1, E2, B
AX-110471201	2A	727,005,198	5.54–7.85	8.84–13.19	E1, E2, B
AX-110130904	2A	727,141,584	5.54–7.85	8.84–13.19	E1, E2, B
AX-109884666	2A	727,169,221	5.51–6.37	10.39–12.77	E1, E2, B
AX-109493384	2A	727,170,336	5.88–7.13	11.01–14.13	E1, E2, B
AX-108792390	2A	727,180,360	5.89–8.42	10.34–15.94	E1, E2, B
AX-111160521	2A	727,181,412	5.67–7.79	8.97–12.77	E1, E2, B
AX-109428070	2A	727,182,816	5.30–6.84	9.14–12.79	E1, E2, B
AX-109292249	2A	727,183,443	5.67–7.79	8.97–12.77	E1, E2, B
AX-111509211	2A	727,183,498	5.67–7.79	8.97–12.77	E1, E2, B
AX-108980791	2A	727,187,444	5.67–7.79	8.97–12.77	E1, E2, B
AX-109961153	2A	727,187,627	6.02–8.35	10.47–15.48	E1, E2, B
AX-108923021	2A	727,190,555	4.73–6.06	7.23–9.55	E1, E2, B
AX-109373105	2A	727,192,289	4.73–6.06	7.23–9.55	E1, E2, B
AX-111512779	2A	727,192,510	4.60–5.81	7.42–10.15	E1, E2, B
AX-111502719	2A	727,192,902	4.73–6.06	7.23–9.55	E1, E2, B
AX-109529880	2A	727,195,932	4.73–6.06	7.23–9.55	E1, E2, B
AX-110447468	2A	727,198,143	4.96–6.45	7.58–9.55	E1, E2, B
AX-108980644	2A	727,243,309	4.76–6.17	8.97–12.30	E1, E2, B
AX-110362101	2A	727,243,960	4.73–6.06	7.23–9.55	E1, E2, B
AX-109360792	2A	727,245,925	5.64–7.47	8.66–11.79	E1, E2, B
AX-110397450	2A	727,285,210	4.73–6.06	7.23–9.55	E1, E2, B
AX-108838121	2A	727,614,295	5.17, 5.79	7.95, 9.59	E2, B
AX-108908177	2A	727,639,917	5.14, 5.90	7.71, 8.99	E2, B
AX-109430725	2A	727,658,142	5.14, 5.90	7.71, 8.99	E2, B
AX-109290174	2A	727,955,888	4.70–6.44	8.14–11.94	E1, E2, B
AX-111064107	2A	728,020,754	4.81, 5.05	7.40, 9.45	E1, B
AX-111727771	2A	728,020,803	4.64–6.35	8.14–11.94	E1, E2, B
AX-109884043	2A	728,026,513	4.64–6.35	8.14–11.94	E1, E2, B
AX-108928895	2A	728,029,569	5.50, 5.89	8.43, 9.42	E2, B
AX-111628949	2A	728,070,767	5.43, 5.97	8.30, 9.34	E2, B
AX-111044883	2A	728,072,564	5.48, 6.02	8.35, 9.35	E2, B
AX-110624913	2A	728,073,136	5.43, 5.97	8.30, 9.34	E2, B
AX-109973650	2A	728,132,521	4.64–6.35	8.14–11.94	E1, E2, B
AX-111609105	7B	611,944,380	4.76, 4.97	5.76, 7.33	E2, B
AX-111031595	7B	611,960,672	4.77, 4.96	5.81, 7.34	E2, B
AX-109440948	7D	611,243,842	4.70, 5.07	10.95, 12.17	E1, B
AX-94482751	7D	611,586,124	4.59, 4.72	12.81, 13.88	E1, B

E1: 2019, E2: 2020, B: BLUP of 2 years, and PVE, phenotypic variance explained.

### Prediction of Candidate Genes for Grain Mo Concentration in Wheat

The genomic regions of 500 kb upstream and downstream of the 52 common significant SNPs were defined as candidate regions and used to explore the candidate genes for GMoC. Genes in three candidate regions on chromosomes 2A, 7B, and 7D were screened, which were located in the intervals of 726.26–728.63 Mb (2A), 611.44–612.46 Mb (7B), and 610.74–612.09 Mb (7D). The potential candidate genes were selected based on their annotation information and functions of the homologous genes in *Arabidopsis* and *Oryza sativa*. Finally, three potential candidate genes associated with GMoC were screened out, which were all distributed in the candidate region of chromosome 2A, including TraesCS2A02G496200, TraesCS2A02G496700, and TraesCS2A02G497200 at the position of 727,244,098–727,245,793 bp, 727,914,334–727,918,774 bp, and 728,067,465–728,072,317 bp, respectively (Table 3).

**Table 3:** Potential candidate genes underlying GMoC trait in wheat.

Gene ID	Chr.	Pos. (bp)	At Ortholog	Os Ortholog	Gene annotation
TraesCS2A02G496200	Chr2A	727,244,098–727,245,793	AT1G80310	LOC_Os01g45830	Molybdate transporter 1;2
TraesCS2A02G496700	Chr2A	727,914,334–727,918,774	AT2G25680	LOC_Os08g01120	Molybdate transporter 1;1
TraesCS2A02G497200	Chr2A	728,067,465–728,072,317	AT5G20990	LOC_Os04g56610	Molybdopterin biosynthesis protein CNX1

The gene TraesCS2A02G496200 encodes a molybdate transporter 1;2 protein associated with GMoC and was only 132 bp from the significant SNP marker AX-109360792 (Chr 2A: 727,245,925 bp). The gene TraesCS2A02G496700 is annotated as a molybdate transporter 1;1 associated with the GMoC and was close (37.1 kb) to the SNP marker AX-109290174 (Chr 2A: 727,955,888 bp). The SNP marker AX-111628949 (Chr 2A: 728,070,767 bp) on chromosome 2A was located in the intron of the TraesCS2A02G497200 gene encoding a molybdopterin biosynthesis protein CNX1, and the SNP marker AX-111044883 (Chr 2A: 728,072,564 bp) was close (247 bp) to the 5′-untranslated region.

**Figure 4:**
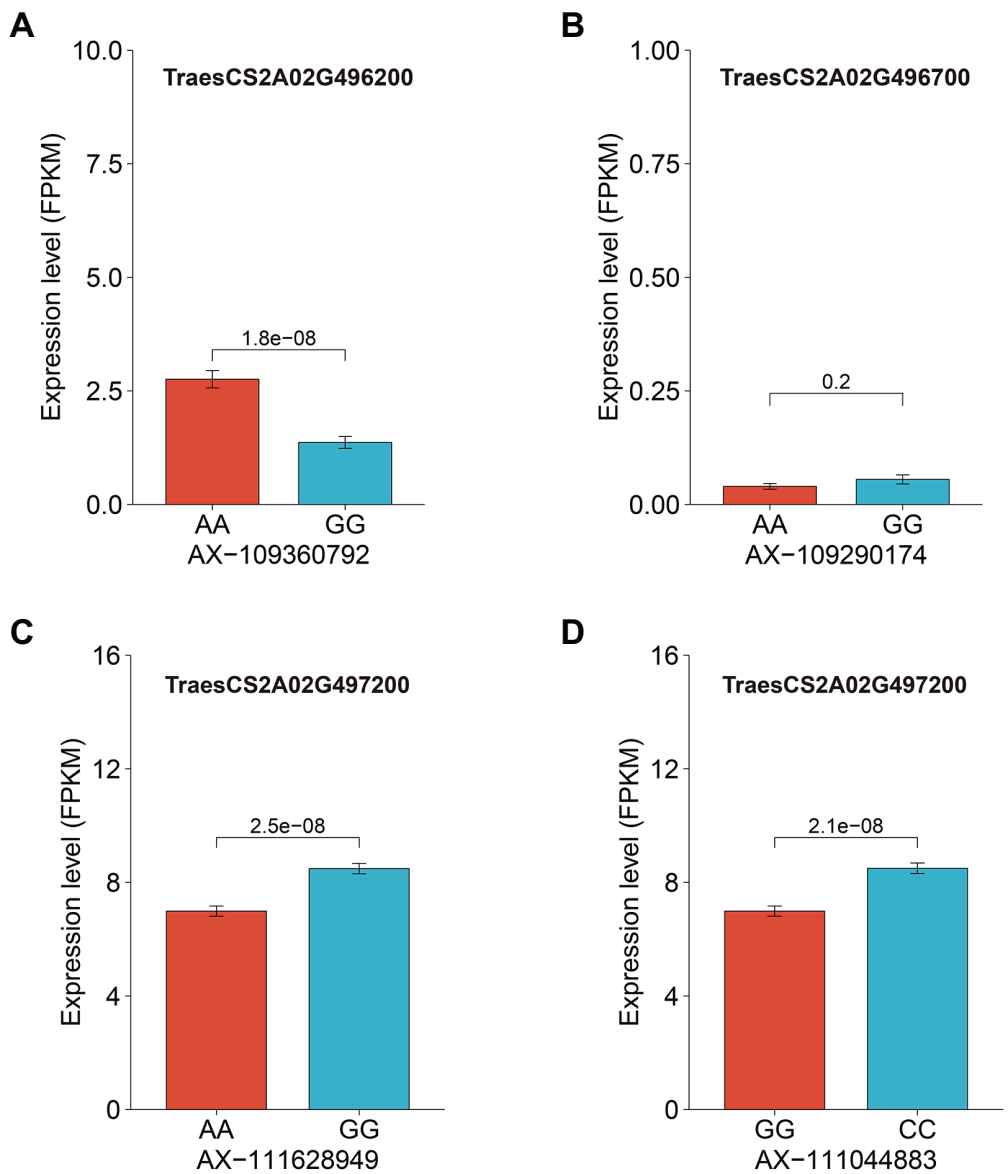
The average expression levels of three candidate genes of wheat accessions with different genotypes of its closely associated SNPs. **(A)**: The expression level of TraesCS2A02G496200 between accessions with different genotypes at SNP AX-109360792; **(B)**: The expression level of TraesCS2A02G496700 between accessions with different genotypes at SNP AX-109290174; and **(C,D)**: The expression level of TraesCS2A02G497200 between accessions with different genotypes at SNPs AX-111628949 and AX-111044883.

We then analyzed the expression levels of these candidate genes in grains at 20 days after pollination in 207 wheat accessions. Surprisingly, TraesCS2A02G496700 showed extremely low expression in grains (Supplementary Figure 3) and exhibited no significant difference in expression among wheat accessions carrying different genotypes of the SNP AX-109290174 (Figure 4B; Supplementary Table 4). In contrast, both TraesCS2A02G496200 (AX-109360792) and TraesCS2A02G497200 (AX-111628949 and AX-111044883) showed significant differences in expression among different genotypes of the closely associated SNPs (Figure 4; Supplementary Table 4). GMoC tended to be consistent with TraesCS2A02G496200 expression at the AX-109360792 site and inverse to TraesCS2A02G497200 expression at the AX-111628949 and AX-111044883 site (Figure 4; Supplementary Figure 2).

## Discussion

As an essential element, Mo is indispensable for nearly all living organisms (Mendel and Bittner, 2006). Several genes have been identified to be responsible for natural variations of Mo level in plants (Huang and Salt, 2016; Yang et al., 2018; Huang et al., 2019; Whitt et al., 2020). However, the genetic basis for natural variation of Mo level in wheat is still poorly understood. Thus, we carried out a GWAS analysis on the Mo concentration in grains among 207 wheat accessions grown in two consecutive years to dissect the genetic basis for Mo accumulation in wheat grains.

### Wide Variations of Mo Concentration in Wheat Grains

When grown in acidic soils, wheat tends to have low grain yield, quality, and Mo content due to Mo efficiency (Yu et al., 1999; Chatterjee and Nautiyal, 2001; Modi, 2002; Kaiser et al., 2005). Compared with the application of Mo fertilizer, the application of wheat seeds with high Mo concentrations is more economical and environment-friendly to solve the problem of Mo deficiency in acidic soils (Brennan and Bolland, 2007). Therefore, breeding of wheat cultivars with high GMoC may be an effective approach to overcome the Mo deficiency for wheat in acidic soils. Our results showed that GMoC had great variations among the 207 wheat accessions, ranging from 293.36 to 1324.41 ng/g in 2019 and from 496.00 to 1941.58 ng/g in 2020 (Table 1). There was a moderate heritability (*H*^2^ = 0.497) between the 2 years, suggesting that GMoC is affected by both genetic and environmental factors. This seems to be consistent with previous findings in *Arabidopsis* (Baxter et al., 2008). Norton et al. (2014) and Yang et al. (2018) reported an even higher heritability of Mo accumulation in rice grains. Our results revealed that the GMoC between 2019 and 2020 was significantly positively correlated with each other (*r* = 0.621; *p* < 0.001), indicating that GMoC is relatively stable across different years and genetic factors are important determinants. Therefore, the wheat varieties with stably high GMoC like Taishan 5, Xinmai 18, and Bainong 160 have the potential to be utilized in future wheat breeding programs.

### Identification of Potential Candidate Genes for Grain Mo Concentration in Wheat

The bi-parental QTL mapping in a previous study identified one QTL (*Qgmo.tamu.3B.540*) associated with GMoC on chromosome 3B (Yu et al., 2021). However, no GMoC-related genes have been identified by GWAS in common wheat so far. In the present study, we identified 52 common SNPs significantly associated with GMoC through GWAS, which are distributed on chromosome 2A, 7B, and 7D. Interestingly, 48 out of the 52 common SNPs were located on chromosome 2A and distributed in the region of 726,761,412–728,132,521 bp. Based on gene functional annotations, TraesCS2A02G496200, TraesCS2A02G496700, and TraesCS2A02G497200 on chromosome 2A were identified as potential candidate genes.

TraesCS2A02G496700 and TraesCS2A02G496200, which are annotated as molybdate transport 1;1 and molybdate transport 1;2, respectively, are two specific molybdate transporters and belong to the Molybdate Transporter 1 (MOT1) family. In eukaryotes, the MOT1 family mediates high-affinity and specific molybdate transport (Tejada-Jimenez et al., 2013). The orthologous genes of *MOT1* have been identified in different species, such as *Chlamydomonas reinhardtii* (Tejada-Jimenez et al., 2007), *A. thaliana* (Tomatsu et al., 2007; Baxter et al., 2008; Gasber et al., 2011), rice (Norton et al., 2014; Yang et al., 2018; Huang et al., 2019; Wang et al., 2020; Ishikawa et al., 2021), maize (Asaro et al., 2016), strawberry (Liu et al., 2020b), *Lotus japonicus* (Gao et al., 2016; Duan et al., 2017), and *Medicago truncatula* (Tejada-Jimenez et al., 2017; Gil-Diez et al., 2019). The phylogenetic tree showed that TraesCS2A02G496700 is the homolog of LOC_Os08g01120 (*OsMOT1;1*) in rice, while TraesCS2A02G496200 is the homolog of LOC_Os01g45830 (*OsMOT1;2*) in rice and AT1G80310 (*AtMOT1;2*) in *Arabidopsis* (Supplementary Figure 4). MOT1 is responsible for molybdate uptake, translocation, and accumulation (Tomatsu et al., 2007; Baxter et al., 2008; Gasber et al., 2011; Tejada-Jimenez et al., 2013). Yang et al. (2018) attributed the variations of Mo accumulation in rice grains to changes in the expression level of *OsMOT1;1*. However, TraesCS2A02G496700 showed quite low expression in wheat grains (Supplementary Figure 3), which could be verified by the public transcript data from expVIP platform (Supplementary Figure 5). The public data demonstrate that TraesCS2A02G496700 is highly expressed in roots. Huang et al. (2019) revealed that the expression level of *OsMOT1;1* in roots affects the Mo concentration in rice grains. Therefore, TraesCS2A02G496700 is considered as a candidate gene in this study.

In *A. thaliana*, AtMOT1;2 (formerly named AtMOT2) is a vacuolar molybdate transporter and involved in inter-organ Mo translocation as well as Mo accumulation in seeds (Gasber et al., 2011). Ishikawa et al. (2021) identified OsMOT1;2 as a vacuolar molybdate export protein that plays an important role in inter-organ Mo distribution in rice. The deletion of *OsMOT1;2* decreased the grain Mo concentration in the rice mutant *osmot1;2*. In our study, TraesCS2A02G496200 is considered as the ortholog of MOT1;2 in wheat (Table 3; Supplementary Figure 4). At the AX-109360792 locus (Chr 2A: 727,245,925 bp; close to the upstream of TraesCS2A02G496200), the accessions with the AA genotype had both significantly higher GMoC and TraesCS2A02G496200 expression than those with the GG genotype (Figure 4A; Supplementary Figure 2), suggesting that it is a candidate gene of GMoC. However, the expression of TraesCS2A02G496200 was the highest in leaves and shoots, followed by roots, while the lowest in grains (Supplementary Figure 5). This is similar to the expression pattern of *OsMOT1;2* in rice and *AtMOT1;2* in *A. thaliana*, suggesting its role in inter-organ Mo translocation and distribution (Gasber et al., 2011; Ishikawa et al., 2021). Therefore, it is of great significance to explore the molecular mechanism for the regulatory effect of these two candidate genes (TraesCS2A02G496700 and TraesCS2A02G496200) on Mo accumulation in wheat grains in future studies.

The third potential candidate gene TraesCS2A02G497200 is annotated as molybdopterin biosynthesis protein CNX1 (cofactor for nitrate reductase and xanthine dehydrogenase 1), which is involved in Moco biosynthesis in plants by inserting Mo into molybdopterin (Schwarz et al., 2000; Kuper et al., 2003; Llamas et al., 2004, 2006; Tejada-Jimenez et al., 2018). Significant differences in phenotype and expression were observed for the genotypes at two SNPs (AX-111628949 and AX-111044883) closely associated with TraesCS2A02G497200. Interestingly, GMoC tended to have an inverse relationship with the gene expression at the two sites (Figure 4; Supplementary Figure 2). This phenomenon may be attributed to the conversion of more molybdate into Moco under the catalysis of CNX1, which leads to a decrease in GMoC. CNX1 has been found to be constitutively expressed in all organs of *Arabidopsis* plants (Schwarz et al., 2000). Similarly, the expression of TraesCS2A02G497200 was also found in almost all organs of wheat plants and was higher in roots than in grains (Supplementary Figure 5). This might be due to a large amount of Mo absorbed by roots from the soil, which is then used for MoCo synthesis. Thus, the above-mentioned three genes are considered as potential candidate genes for GMoC in wheat, which may be utilized in wheat breeding. However, further functional studies are required to verify their functions.

## Data Availability Statement

The datasets presented in this study can be found in online repositories. The names of the repository/repositories and accession number(s) can be found in the article/Supplementary Material.

## Author Contributions

ZfZ, ZL, and ZW designed the experiment. CL conducted the field experiment. ZjZ and YP investigated the phenotype and revised and edited the manuscript. XJ and SY conducted the GWAS analysis. XJ and YP drafted the manuscript. All authors have read and approved the final manuscript.

## Funding

This research was funded by National Natural Science Foundation of China (31701506), the key area projects of Guizhou education department (KY [2020]044), the Agriculture Research System of Henan Province (S2010-01), the Science-Technology Foundation for Excellent Youth Scholars of Henan Academy of Agricultural Sciences (2020YQ02), and Special fund projects of science and technology development of Henan Academy of Agricultural Sciences (YNK20177511).

## Conflict of Interest

The authors declare that the research was conducted in the absence of any commercial or financial relationships that could be construed as a potential conflict of interest.

## Publisher’s Note

All claims expressed in this article are solely those of the authors and do not necessarily represent those of their affiliated organizations, or those of the publisher, the editors and the reviewers. Any product that may be evaluated in this article, or claim that may be made by its manufacturer, is not guaranteed or endorsed by the publisher.

## Footnotes

^1^ https://github.com/YinLiLin/CMplot

^2^ http://www.wheat-expression.com/
