# Supplementary material for: Genome-Wide Association Study Reveals Genomic Regions Associated With Molybdenum Accumulation in Wheat Grains
Source: Front Plant Sci. 2022 Mar 2;13:854966. doi: 10.3389/fpls.2022.854966 (PMC8924584; doi:10.3389/fpls.2022.854966)
Supplement: Supplementary file 1 [file Data_Sheet_1.docx]

Supplementary Material

**Supplementary Figures**


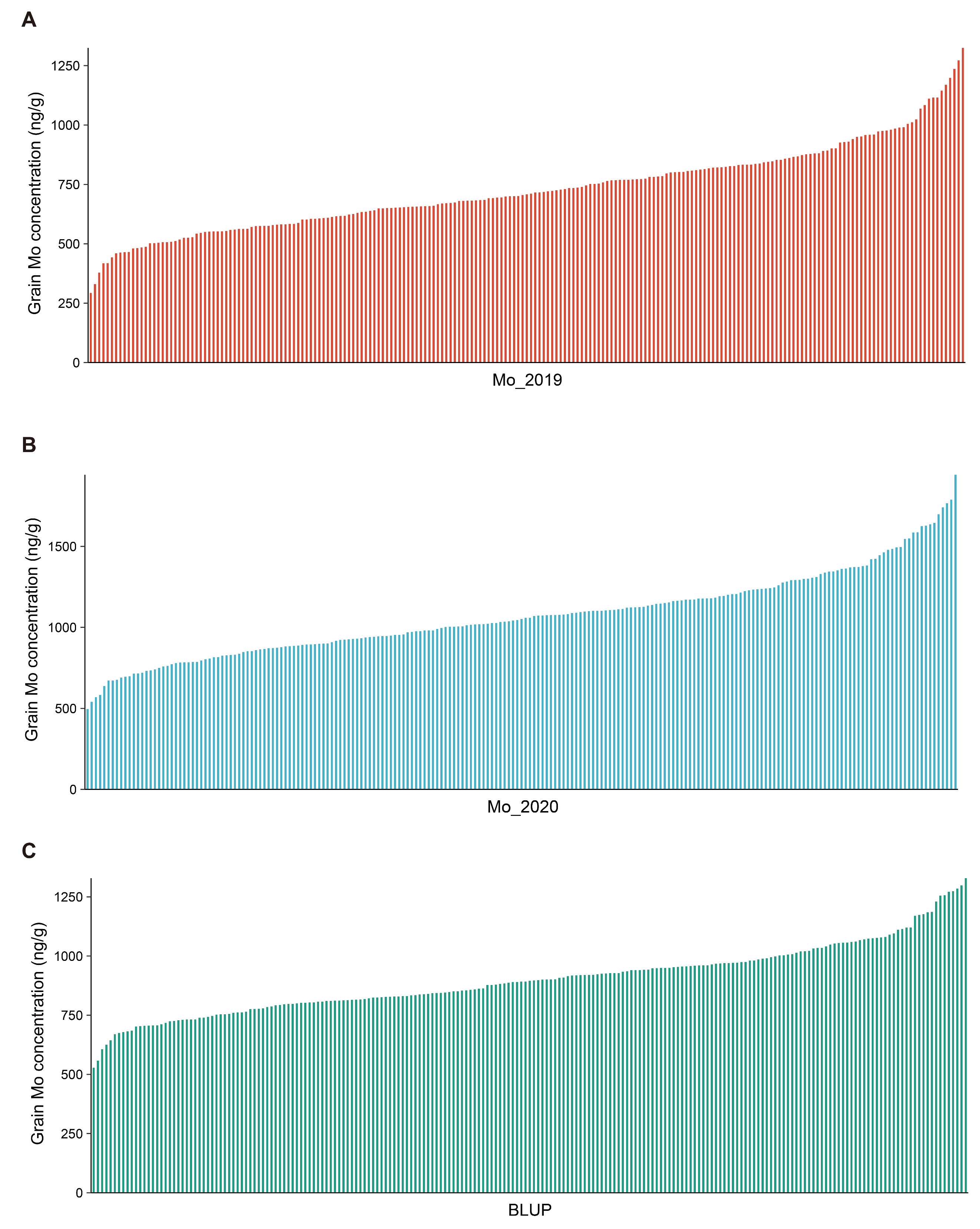


**Supplementary Figure 1.** Grain Mo concentration (ng/g) distribution of the wheat association panel in two years (2019/2020) and BLUP values.


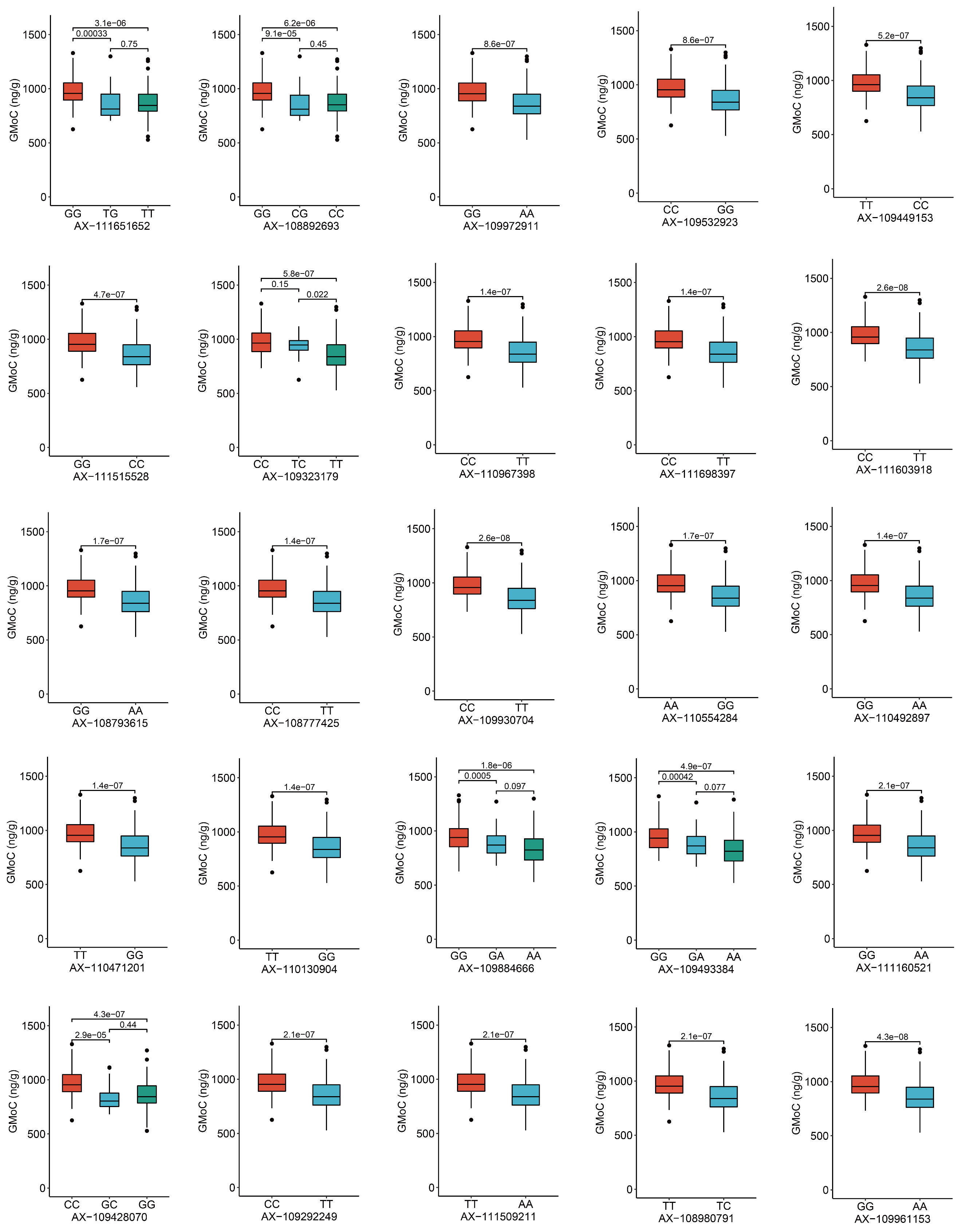


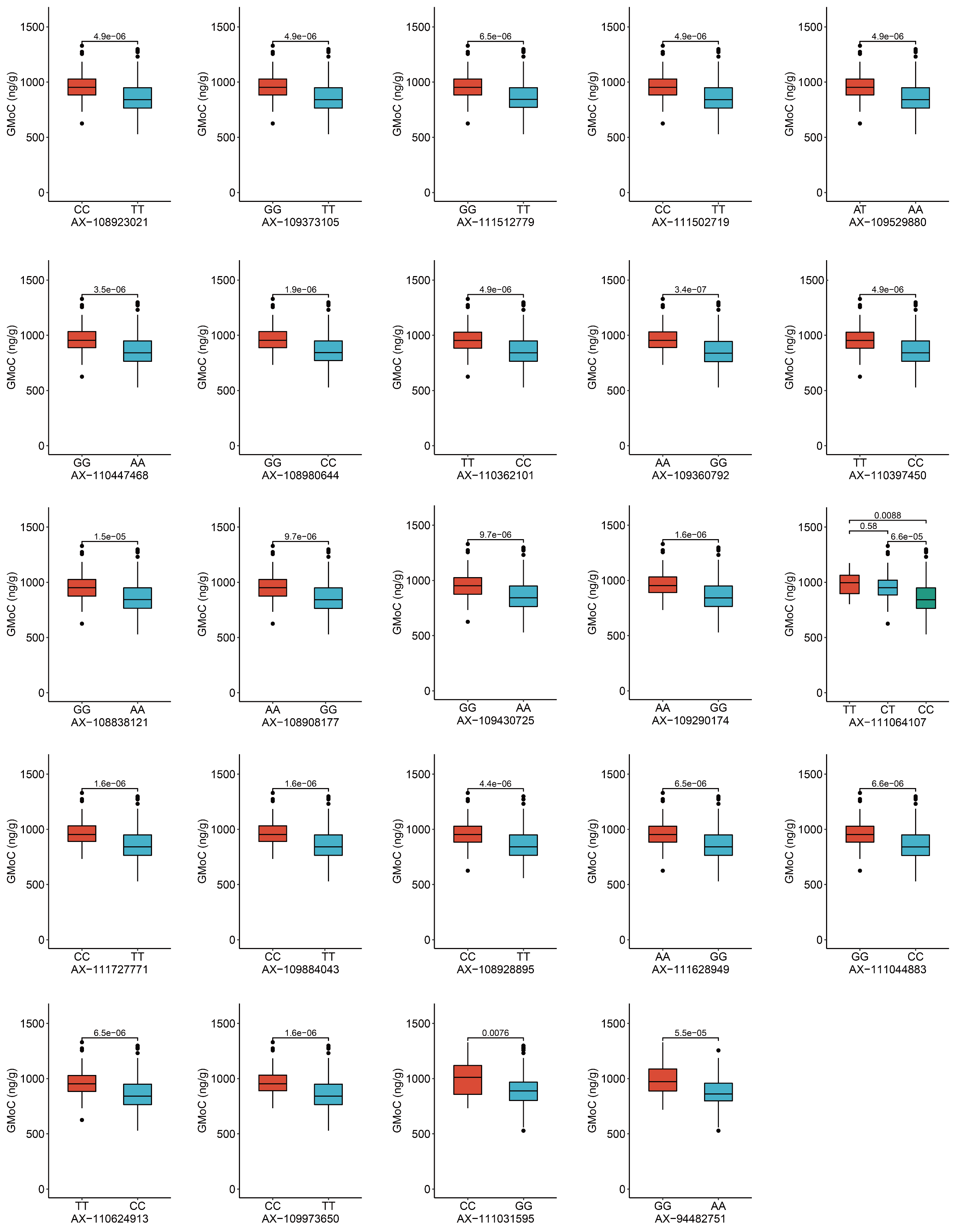


**Supplementary Figure 2.** Phenotypic differences in grain Mo concentration of wheat accessions carrying different genotypes of 49 common SNPs.


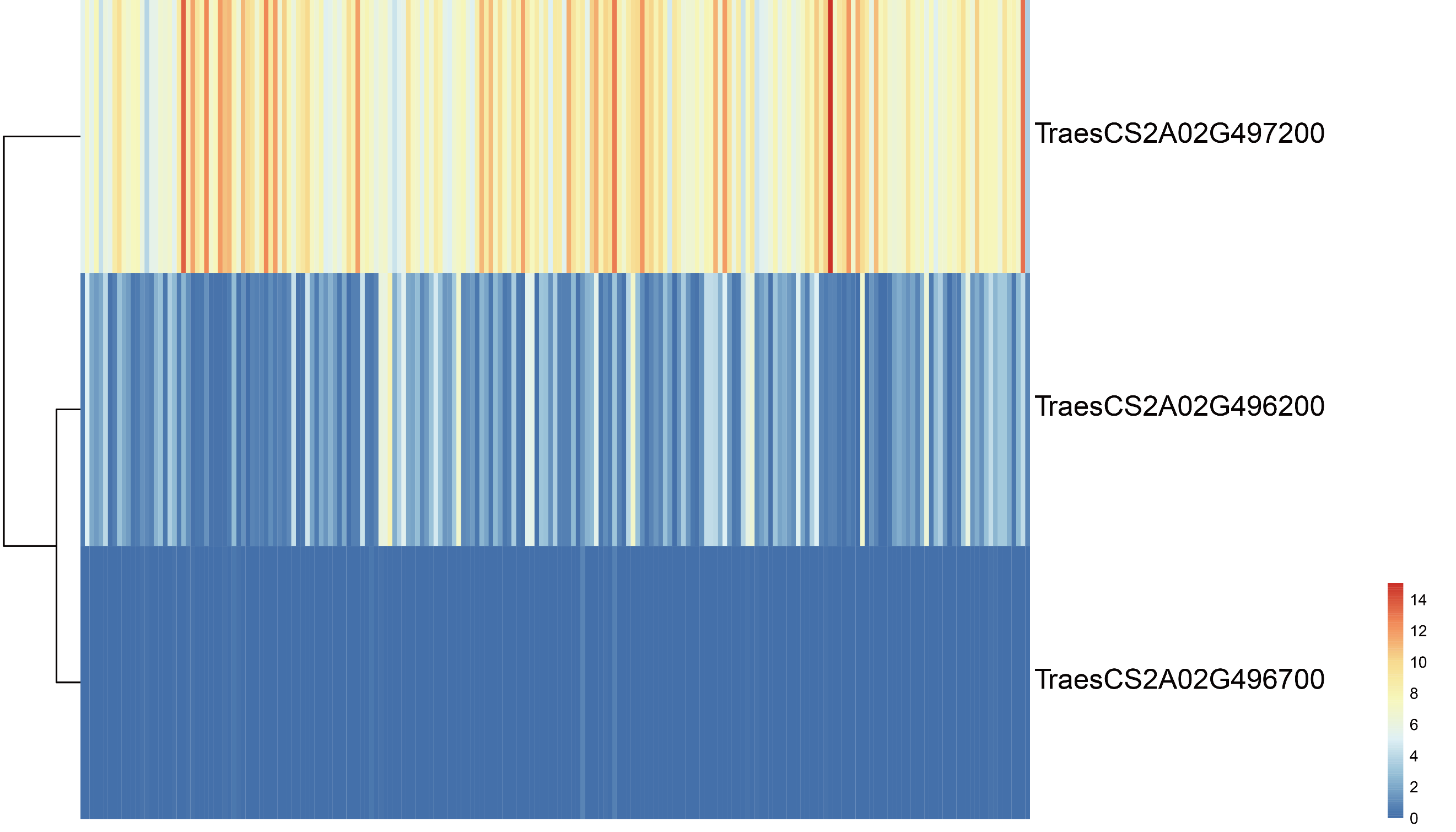


**Supplementary Figure 3.** Heatmap of expression levels (FPKM) of the three candidate genes in the grains of association panel.


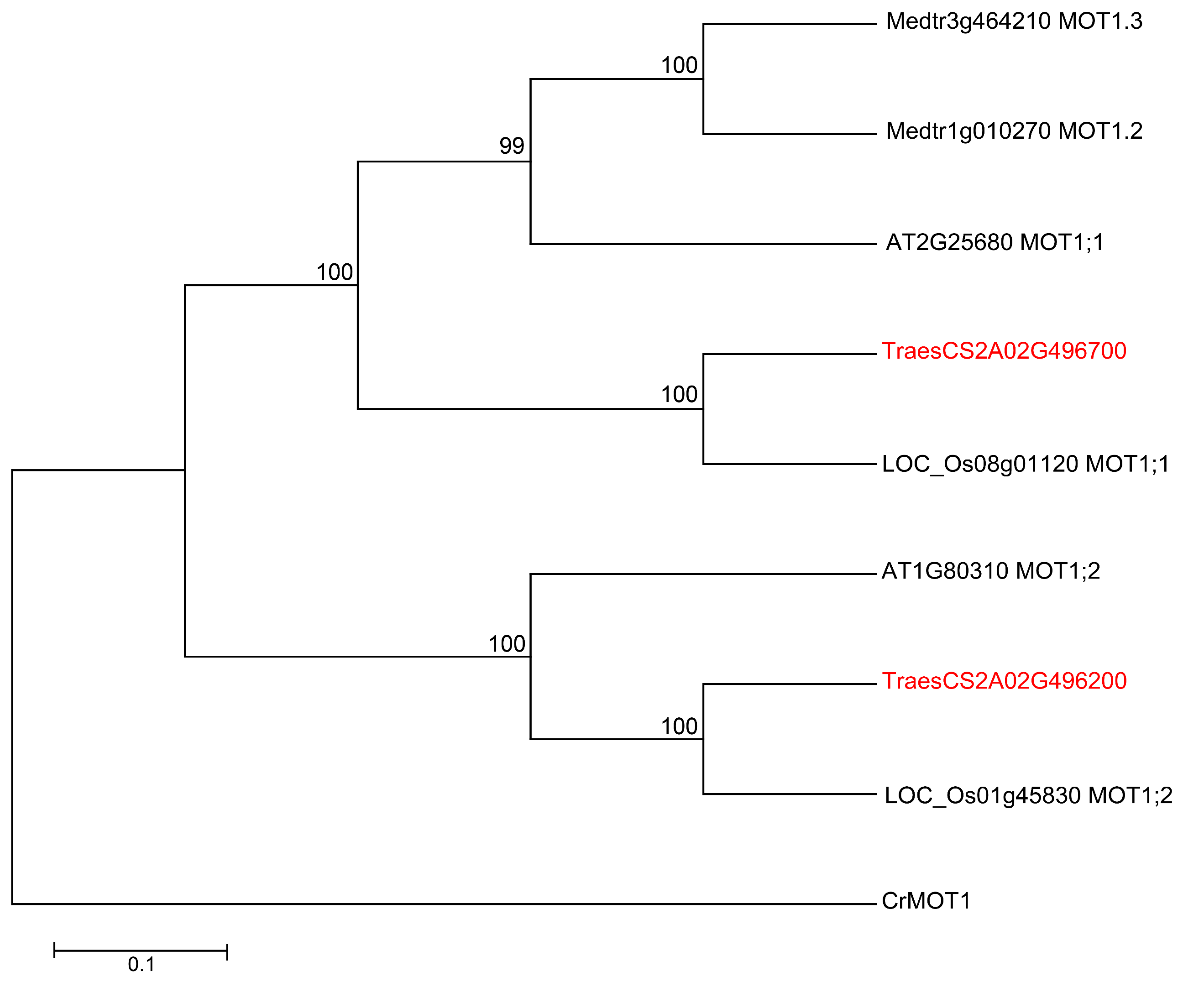


**Supplementary Figure 4.** Phylogenetic analysis of MOT1 in wheat and other species. The number above the branch represented the bootstrap value. Medtr3g464210 (MtMOT1.3) and Medtr1g010270 (MtMOT1.2) from *Medicago truncatula*; AT2G25680 (AtMOT1;1) and AT1G80310 (AtMOT1;2) from *Arabidopsis thaliana*; LOC_Os08g01120 (OsMOT1;1) and LOC_Os01g45830 (OsMOT1;2) from *Oryza sativa*; CrMOT1 from *Chlamydomonas reinhardtii*; TraesCS2A02G496700 and TraesCS2A02G496200 from *Triticum aestivum*.


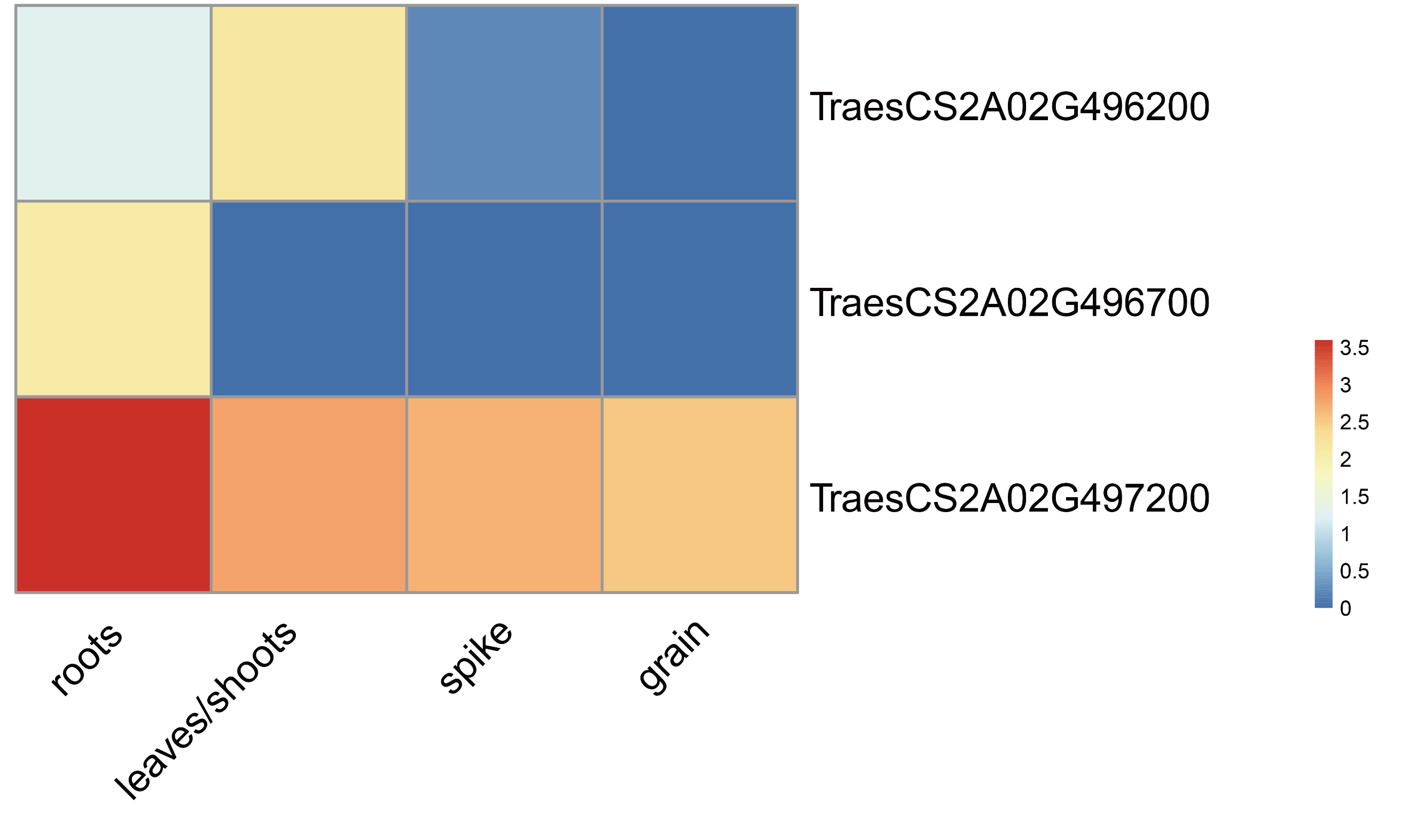


**Supplementary Figure 5.** Heatmap of expression levels (log_2_TPM) of the three potential candidate genes for GMoC in different wheat tissues.

**Supplementary Tables**

**Supplementary Table 1.** Grain Mo concentration of the association panel planted in two years (2019 and 2020) and BLUP values.

| **Accession ID** | **Variety name** | **Mo_2019 (ng/g)** | **Mo_2020 (ng/g)** | **BLUP (ng/g)** |
| --- | --- | --- | --- | --- |
| W1 | Changwu 135 | 767.32 | 1026.59 | 898.06 |
| W2 | Shan 229 | 836.30 | 1146.70 | 967.70 |
| W3 | Xiaoyan 6 | 562.31 | 870.32 | 765.02 |
| W4 | Lantian 10 | 844.94 | 1144.72 | 970.15 |
| W5 | Luohan 3 | 814.35 | 1037.38 | 919.36 |
| W6 | Jing 411 | 858.23 | 1104.14 | 960.10 |
| W7 | Ningdong 1 | 890.71 | 1102.54 | 971.47 |
| W8 | Mianyang 11 | 502.77 | 758.67 | 701.98 |
| W9 | Taishan 5 | 1236.45 | 1644.87 | 1298.51 |
| W10 | Xinmai 13 | 1169.48 | 1362.52 | 1169.87 |
| W11 | Xinmai 18 | 1116.00 | 1698.07 | 1273.74 |
| W12 | Zhoumai 16 | 843.14 | 894.69 | 877.41 |
| W13 | Yuanfeng 139 | 629.80 | 1059.24 | 859.45 |
| W14 | Fengchan 3 | 800.39 | 1420.28 | 1055.22 |
| W15 | Zhongyu 8 | 708.26 | 1223.48 | 948.82 |
| W16 | Bainong 160 | 1198.41 | 1766.23 | 1329.19 |
| W17 | Luomai 21 | 972.83 | 1306.43 | 1076.80 |
| W18 | Yanzhan 4110 | 892.70 | 1016.17 | 940.40 |
| W19 | Han 6172 | 753.48 | 898.18 | 845.68 |
| W20 | Huaimai 21 | 1115.50 | 1098.16 | 1052.64 |
| W21 | Mianyang 26 | 960.08 | 1170.63 | 1022.09 |
| W22 | Lunxuan 715 | 752.26 | 740.56 | 787.18 |
| W23 | Youpi 1 | 574.70 | 695.63 | 705.25 |
| W24 | Xifeng 9 | 781.58 | 825.03 | 829.09 |
| W25 | Nongda 198 | 878.25 | 1076.85 | 957.42 |
| W26 | Kedong 81 | 1144.58 | 1126.33 | 1073.72 |
| W27 | Linfen 10 | 1324.41 | 1483.43 | 1271.45 |
| W28 | Fengkang 5 | 853.37 | 1371.77 | 1056.86 |
| W29 | Changfeng 1 | 563.13 | 1215.16 | 892.31 |
| W30 | Jingdong 1 | 837.55 | 1740.15 | 1186.70 |
| W31 | Jinmai 21 | 551.87 | 969.39 | 797.66 |
| W32 | Jimai 23 | 928.54 | 1228.90 | 1031.94 |
| W33 | Hanxuan 10 | 650.85 | 1282.41 | 949.38 |
| W34 | Xianshixinmai | 652.81 | 749.69 | 753.93 |
| W35 | Chixiaomai | 769.54 | 980.66 | 881.97 |
| W36 | Bima 1 | 1068.76 | 1204.56 | 1074.61 |
| W37 | Aimengniu | 671.64 | 940.59 | 831.16 |
| W38 | Wenmai 6 | 506.55 | 1051.64 | 811.26 |
| W39 | Mazhamai | 511.84 | 675.71 | 674.77 |
| W40 | Dayuhua | 767.09 | 1107.11 | 927.63 |
| W41 | Chushanbao | 587.59 | 715.78 | 717.42 |
| W42 | Dalibanmang | 580.54 | 762.31 | 731.96 |
| W43 | Baimazha | 293.36 | 496.00 | 528.13 |
| W44 | Ganmai 8 | 694.70 | 908.84 | 827.96 |
| W45 | Yunmai 34 | 880.87 | 1111.33 | 971.09 |
| W46 | Wuyimai | 480.04 | 807.77 | 711.69 |
| W47 | Nanda 2419 | 853.46 | 946.83 | 900.41 |
| W48 | Norin 10 | 648.82 | 971.94 | 834.30 |
| W49 | Hanyangmai | 730.40 | 1073.41 | 901.71 |
| W50 | Lovrin 10 | 990.94 | 1381.97 | 1111.28 |
| W51 | Aifeng 3 | 769.53 | 928.91 | 862.91 |
| W52 | Yunhan 618 | 583.77 | 779.44 | 739.46 |
| W53 | Yannong 21 | 655.99 | 891.48 | 807.31 |
| W54 | Jinmai 47 | 764.39 | 1236.95 | 974.45 |
| W55 | Changwu 58 | 846.99 | 1276.73 | 1019.52 |
| W56 | Zhenghan 1 | 680.76 | 782.95 | 776.47 |
| W57 | Chang 6878 | 700.60 | 847.49 | 807.54 |
| W58 | Luohan 1 | 805.92 | 1042.18 | 918.02 |
| W59 | Luohan 6 | 551.36 | 1081.74 | 838.84 |
| W60 | Luohan 11 | 543.13 | 671.13 | 684.60 |
| W61 | PuXing 5 | 985.05 | 1177.87 | 1033.96 |
| W62 | Ruiquanmai 24 | 784.38 | 882.52 | 851.29 |
| W63 | Dehongfu 2 | 833.17 | 1005.87 | 914.68 |
| W64 | Zhongmai 895 | 951.73 | 922.35 | 927.59 |
| W65 | Huaimai 35 | 700.82 | 943.48 | 842.97 |
| W66 | Longpingmai 518 | 1083.79 | 1178.83 | 1070.67 |
| W67 | Huaimai 30 | 609.37 | 953.28 | 812.90 |
| W68 | Zhongyuan 6 | 633.91 | 872.22 | 792.09 |
| W69 | Pingan 8 | 716.27 | 852.53 | 815.16 |
| W70 | Bainong 207 | 950.13 | 980.26 | 948.32 |
| W71 | Cun 1 | 980.33 | 1075.99 | 994.70 |
| W72 | Zhoumai 26 | 771.81 | 784.49 | 810.56 |
| W73 | Luomai 18 | 771.00 | 787.33 | 811.31 |
| W74 | Baofeng 10-82 | 739.44 | 1003.71 | 879.37 |
| W75 | Su 553 | 833.31 | 803.24 | 840.11 |
| W76 | 09N37 | 601.82 | 671.74 | 706.44 |
| W77 | Guinong 17 | 607.94 | 714.41 | 724.41 |
| W78 | Hengguan 35 | 669.83 | 1069.50 | 877.96 |
| W79 | Yumai 18 | 552.71 | 932.96 | 784.55 |
| W80 | Yumai 13 | 652.49 | 1021.76 | 854.00 |
| W81 | Yumai 47 | 727.78 | 937.68 | 850.76 |
| W82 | Zhengmai 004 | 958.93 | 1059.41 | 980.71 |
| W83 | Yumai 50 | 820.36 | 827.03 | 844.11 |
| W84 | Taikong 6 | 487.28 | 719.40 | 681.81 |
| W85 | Huapei 5 | 873.21 | 1170.68 | 990.12 |
| W86 | Hua 9987 | 821.50 | 1075.27 | 935.94 |
| W87 | Yujiao 5 | 657.50 | 946.47 | 828.12 |
| W88 | Yunong 416 | 860.68 | 1205.79 | 998.43 |
| W89 | Bainong 64 | 930.03 | 1293.28 | 1056.19 |
| W90 | Zhoumai 9 | 562.46 | 1032.80 | 824.91 |
| W91 | Zhoumai 13 | 658.88 | 874.44 | 802.10 |
| W92 | Zhoumai 8425B | 625.30 | 896.68 | 797.92 |
| W93 | Kaimai 21 | 722.83 | 815.54 | 803.96 |
| W94 | Luozhen 1 | 681.57 | 926.06 | 829.47 |
| W95 | Neixiang 188 | 822.01 | 1121.60 | 953.19 |
| W96 | Aiyou 26-2 | 694.95 | 1076.18 | 889.68 |
| W97 | Changgeheili | 570.94 | 853.47 | 761.99 |
| W98 | Lvmai 1 | 802.44 | 1018.49 | 908.01 |
| W99 | Subeimai 1 | 545.83 | 877.30 | 761.52 |
| W100 | Jinan 17 | 682.60 | 887.78 | 815.75 |
| W101 | Jimai 20 | 462.91 | 734.64 | 678.45 |
| W102 | Shan 225 | 704.83 | 1259.26 | 960.73 |
| W103 | Xinong 979 | 801.35 | 1183.49 | 968.37 |
| W104 | Baxter | 605.14 | 859.77 | 776.91 |
| W105 | CD87 | 559.57 | 837.27 | 751.84 |
| W106 | Kukri | 418.62 | 582.54 | 606.13 |
| W107 | Faguomai | 465.45 | 637.80 | 643.72 |
| W108 | Belero | 484.53 | 568.72 | 625.31 |
| W109 | Fundulea 900 | 582.07 | 690.66 | 706.14 |
| W110 | Tincurrin | 635.12 | 698.19 | 728.44 |
| W111 | H149 | 725.65 | 885.77 | 830.86 |
| W112 | FaB08 | 552.16 | 773.03 | 725.45 |
| W113 | FaB20 | 606.84 | 731.79 | 730.41 |
| W114 | Bainong 416 | 660.25 | 1299.18 | 959.02 |
| W115 | Luo 31 | 623.14 | 1133.97 | 884.51 |
| W116 | Taihemai 1 | 718.18 | 1234.55 | 956.55 |
| W117 | Junmai 35 | 680.39 | 1360.89 | 989.16 |
| W118 | Luomai 23 | 673.62 | 956.58 | 837.78 |
| W119 | Jiaomai 266 | 655.03 | 1027.35 | 856.99 |
| W120 | Wennong 14 | 827.42 | 1192.52 | 981.30 |
| W121 | Jimai 22 | 758.12 | 1329.65 | 1006.28 |
| W122 | Ningmai 9 | 443.08 | 899.74 | 731.95 |
| W123 | 10EW28 | 459.86 | 816.09 | 707.32 |
| W124 | 10EW137 | 527.84 | 989.91 | 796.37 |
| W125 | Zhou 18 | 734.57 | 1344.06 | 1002.91 |
| W126 | 04Zhong38 | 692.06 | 1545.44 | 1061.42 |
| W127 | Yumai 34-9901 | 517.92 | 900.41 | 759.75 |
| W128 | Zheng 9023-8 | 482.10 | 784.89 | 704.02 |
| W129 | Zheng 103 | 525.61 | 1240.10 | 887.68 |
| W130 | Calingiri | 554.19 | 829.05 | 746.83 |
| W131 | Zhengmai 366 | 699.21 | 1291.08 | 970.38 |
| W132 | Zhengmai 7698 | 656.46 | 1344.48 | 974.30 |
| W133 | Zhoumai 22 | 329.94 | 540.59 | 558.02 |
| W134 | Zhoumai 27 | 617.99 | 917.39 | 802.86 |
| W135 | Zhoumai 30 | 716.55 | 1193.57 | 940.86 |
| W136 | Zhoumai 32 | 654.28 | 1201.27 | 920.76 |
| W137 | Liangxing 66 | 464.66 | 1107.85 | 816.53 |
| W138 | Emai 25 | 417.63 | 924.21 | 731.59 |
| W139 | Ningchun 5 | 506.94 | 894.03 | 753.36 |
| W140 | Shannong 33 | 648.78 | 1636.26 | 1078.92 |
| W141 | Zhongmai 175 | 558.07 | 981.42 | 804.37 |
| W142 | Gaocheng 8901 | 502.46 | 1352.12 | 920.41 |
| W143 | Shannongtedali 1 | 796.23 | 1493.49 | 1080.65 |
| W144 | Changmai 9 | 524.99 | 1072.91 | 825.89 |
| W145 | Yangfumai 2 | 504.48 | 1003.93 | 792.93 |
| W146 | Wanmai 53 | 768.98 | 1627.67 | 1120.03 |
| W147 | Fanmai 8 | 666.69 | 1124.71 | 897.14 |
| W148 | Yunmai 51 | 378.61 | 795.78 | 669.92 |
| W149 | Yunmai 53 | 957.67 | 1585.19 | 1173.87 |
| W150 | Yunmai 47 | 508.59 | 864.52 | 743.10 |
| W151 | Xinong 928 | 641.34 | 1233.20 | 927.76 |
| W152 | Xinmai 26 | 583.80 | 1112.89 | 862.26 |
| W153 | Wanmai 47 | 701.03 | 1100.53 | 900.88 |
| W154 | Yangmai 13 | 812.21 | 1034.85 | 917.64 |
| W155 | Gaoyou 2018 | 752.23 | 1018.86 | 889.66 |
| W156 | Gaoyou 9415 | 1272.35 | 1495.48 | 1256.71 |
| W157 | Shiluan 02-1 | 783.69 | 1078.55 | 923.23 |
| W158 | Zheng 1005 | 989.24 | 1102.45 | 1007.73 |
| W159 | Zheng 1105 | 832.70 | 1019.83 | 919.65 |
| W160 | Zheng 1118 | 1023.08 | 1549.04 | 1184.65 |
| W161 | Zheng 1289 | 810.33 | 1123.80 | 949.70 |
| W162 | Zheng 3093 | 721.17 | 1149.50 | 926.33 |
| W163 | Sanyuehuang | 574.88 | 952.92 | 800.07 |
| W164 | Wuhuatou | 901.29 | 1941.58 | 1284.35 |
| W165 | Tutoumai | 772.62 | 1176.96 | 955.39 |
| W166 | Baisuibai | 658.47 | 1166.38 | 909.46 |
| W167 | Qumangmai | 1110.64 | 1586.59 | 1230.72 |
| W168 | Zhengzhou 6 | 774.23 | 1478.49 | 1067.02 |
| W169 | Zhengyin 1 | 684.04 | 1094.14 | 892.27 |
| W170 | Shengxuan 7 | 549.81 | 1005.28 | 810.12 |
| W171 | Shuangfengshou | 617.00 | 976.48 | 824.25 |
| W172 | Zhengzhou 7 | 613.04 | 1044.84 | 847.97 |
| W173 | Wanchangsui | 638.62 | 1162.48 | 900.71 |
| W174 | Yuanzhu | 901.99 | 1012.87 | 942.61 |
| W175 | Longfumai 4 | 601.73 | 975.43 | 818.24 |
| W176 | Yizheng 8165 | 802.58 | 1171.84 | 964.54 |
| W177 | Taizao 2 | 604.65 | 865.84 | 778.96 |
| W178 | Yumai 17 | 671.11 | 1241.63 | 941.82 |
| W179 | Chuanmai 50 | 745.64 | 1122.60 | 925.44 |
| W180 | Chuanmai 46 | 781.33 | 1153.83 | 950.08 |
| W181 | Chuanmai 44 | 866.45 | 1164.35 | 985.30 |
| W182 | Chuanmai 107 | 681.72 | 1444.89 | 1020.58 |
| W183 | Mianyang 35 | 975.35 | 1423.18 | 1120.72 |
| W184 | Zhengmai 101 | 880.60 | 1300.20 | 1040.54 |
| W185 | Zhengmai 518 | 807.67 | 1137.92 | 953.92 |
| W186 | Aikang 58 | 817.32 | 1089.95 | 939.81 |
| W187 | Shannong 22 | 710.79 | 1178.94 | 933.35 |
| W188 | Nongda 211 | 831.39 | 1246.03 | 1002.47 |
| W189 | Shannong 19 | 650.54 | 996.14 | 843.85 |
| W190 | Shannong 06-278 | 616.24 | 948.44 | 813.65 |
| W191 | Yangmai 18 | 575.32 | 787.23 | 739.21 |
| W192 | Ningmai 17 | 575.08 | 831.15 | 755.30 |
| W193 | Ningmai 13 | 579.02 | 883.69 | 776.10 |
| W194 | Zhenmai 168 | 734.40 | 942.50 | 854.98 |
| W195 | Yangmai 12 | 691.85 | 929.78 | 834.62 |
| W196 | Yangmai 14 | 976.52 | 1787.76 | 1255.41 |
| W197 | Huamai 5 | 736.41 | 1373.04 | 1014.26 |
| W198 | Yangmai 20 | 684.50 | 1102.59 | 895.55 |
| W199 | Yangmai 21 | 582.05 | 1002.54 | 820.98 |
| W200 | Zhengmai 379 | 876.91 | 1088.24 | 961.13 |
| W201 | Zhengmai 05706 | 826.85 | 1337.32 | 1034.41 |
| W202 | Zhengmai 113 | 941.32 | 1291.42 | 1059.67 |
| W203 | Zhengmai 369 | 1011.64 | 1369.88 | 1114.46 |
| W204 | Zhengmai 05871 | 868.16 | 1462.81 | 1095.84 |
| W205 | Xinmai 19 | 926.12 | 1624.86 | 1176.86 |
| W206 | Shannong 26 | 824.01 | 1378.06 | 1048.37 |
| W207 | Shijiazhuang 8 | 1004.80 | 1310.64 | 1090.12 |

**Supplementary Table 2.** Total significant SNPs associated with GMoC in three datasets.

| **Environment** | **SNP** | **Chromosome** | **Position (bp)** | ***P* value** | **PVE** |
| --- | --- | --- | --- | --- | --- |
| E1 | AX-109972911 | 2A | 726,763,641 | 1.15E-05 | 8.88 |
| E1 | AX-109532923 | 2A | 726,763,898 | 1.15E-05 | 8.88 |
| E1 | AX-109449153 | 2A | 726,764,021 | 1.19E-05 | 8.82 |
| E1 | AX-111515528 | 2A | 726,770,069 | 1.17E-05 | 8.02 |
| E1 | AX-109323179 | 2A | 726,771,093 | 3.74E-06 | 8.97 |
| E1 | AX-110967398 | 2A | 726,772,232 | 2.88E-06 | 8.84 |
| E1 | AX-111698397 | 2A | 726,775,869 | 2.88E-06 | 8.84 |
| E1 | AX-111603918 | 2A | 726,776,128 | 1.49E-06 | 10.34 |
| E1 | AX-108793615 | 2A | 726,865,730 | 3.59E-06 | 8.87 |
| E1 | AX-108777425 | 2A | 726,971,223 | 2.88E-06 | 8.84 |
| E1 | AX-109930704 | 2A | 726,988,053 | 1.55E-06 | 10.05 |
| E1 | AX-110554284 | 2A | 726,994,887 | 4.03E-06 | 9.34 |
| E1 | AX-110492897 | 2A | 727,004,090 | 2.88E-06 | 8.84 |
| E1 | AX-110471201 | 2A | 727,005,198 | 2.88E-06 | 8.84 |
| E1 | AX-110130904 | 2A | 727,141,584 | 2.88E-06 | 8.84 |
| E1 | AX-109884666 | 2A | 727,169,221 | 2.62E-06 | 10.39 |
| E1 | AX-109493384 | 2A | 727,170,336 | 1.32E-06 | 11.01 |
| E1 | AX-108792390 | 2A | 727,180,360 | 1.28E-06 | 10.34 |
| E1 | AX-111160521 | 2A | 727,181,412 | 2.15E-06 | 8.97 |
| E1 | AX-109428070 | 2A | 727,182,816 | 5.06E-06 | 9.14 |
| E1 | AX-109292249 | 2A | 727,183,443 | 2.15E-06 | 8.97 |
| E1 | AX-111509211 | 2A | 727,183,498 | 2.15E-06 | 8.97 |
| E1 | AX-108980791 | 2A | 727,187,444 | 2.15E-06 | 8.97 |
| E1 | AX-109961153 | 2A | 727,187,627 | 9.55E-07 | 10.47 |
| E1 | AX-108923021 | 2A | 727,190,555 | 1.87E-05 | 7.23 |
| E1 | AX-109373105 | 2A | 727,192,289 | 1.87E-05 | 7.23 |
| E1 | AX-111512779 | 2A | 727,192,510 | 2.52E-05 | 7.42 |
| E1 | AX-111502719 | 2A | 727,192,902 | 1.87E-05 | 7.23 |
| E1 | AX-109529880 | 2A | 727,195,932 | 1.87E-05 | 7.23 |
| E1 | AX-110447468 | 2A | 727,198,143 | 1.09E-05 | 7.58 |
| E1 | AX-108980644 | 2A | 727,243,309 | 1.74E-05 | 8.97 |
| E1 | AX-110362101 | 2A | 727,243,960 | 1.87E-05 | 7.23 |
| E1 | AX-109360792 | 2A | 727,245,925 | 2.29E-06 | 8.66 |
| E1 | AX-110397450 | 2A | 727,285,210 | 1.87E-05 | 7.23 |
| E1 | AX-109290174 | 2A | 727,955,888 | 1.98E-05 | 8.14 |
| E1 | AX-111064107 | 2A | 728,020,754 | 1.54E-05 | 7.40 |
| E1 | AX-111727771 | 2A | 728,020,803 | 2.28E-05 | 8.14 |
| E1 | AX-109884043 | 2A | 728,026,513 | 2.28E-05 | 8.14 |
| E1 | AX-109973650 | 2A | 728,132,521 | 2.28E-05 | 8.14 |
| E1 | AX-111502582 | 7A | 701,768,603 | 3.05E-06 | 11.55 |
| E1 | AX-94551126 | 7B | 700,832,678 | 2.56E-05 | 11.86 |
| E1 | AX-109440948 | 7D | 611,243,842 | 8.54E-06 | 10.95 |
| E1 | AX-94482751 | 7D | 611,586,124 | 1.91E-05 | 12.81 |
| E1 | AX-108812253 | 7D | 611,586,769 | 2.56E-05 | 11.86 |
| E1 | AX-109300285 | 7D | 611,593,773 | 2.48E-05 | 10.92 |
| E2 | AX-111651652 | 2A | 726,761,412 | 1.46E-05 | 10.47 |
| E2 | AX-108892693 | 2A | 726,762,422 | 2.21E-05 | 10.68 |
| E2 | AX-109972911 | 2A | 726,763,641 | 3.95E-06 | 9.67 |
| E2 | AX-109532923 | 2A | 726,763,898 | 3.95E-06 | 9.67 |
| E2 | AX-109449153 | 2A | 726,764,021 | 5.17E-06 | 9.79 |
| E2 | AX-111515528 | 2A | 726,770,069 | 1.51E-07 | 11.66 |
| E2 | AX-109323179 | 2A | 726,771,093 | 1.14E-08 | 13.32 |
| E2 | AX-110967398 | 2A | 726,772,232 | 5.34E-08 | 12.23 |
| E2 | AX-111698397 | 2A | 726,775,869 | 5.34E-08 | 12.23 |
| E2 | AX-111603918 | 2A | 726,776,128 | 2.04E-08 | 15.07 |
| E2 | AX-108793615 | 2A | 726,865,730 | 5.98E-08 | 12.23 |
| E2 | AX-108777425 | 2A | 726,971,223 | 5.34E-08 | 12.23 |
| E2 | AX-109930704 | 2A | 726,988,053 | 1.78E-08 | 14.13 |
| E2 | AX-110554284 | 2A | 726,994,887 | 7.11E-08 | 12.31 |
| E2 | AX-110492897 | 2A | 727,004,090 | 5.34E-08 | 12.23 |
| E2 | AX-110471201 | 2A | 727,005,198 | 5.34E-08 | 12.23 |
| E2 | AX-110130904 | 2A | 727,141,584 | 5.34E-08 | 12.23 |
| E2 | AX-109884666 | 2A | 727,169,221 | 3.10E-06 | 10.48 |
| E2 | AX-109493384 | 2A | 727,170,336 | 4.54E-07 | 11.99 |
| E2 | AX-108792390 | 2A | 727,180,360 | 1.64E-08 | 15.07 |
| E2 | AX-111160521 | 2A | 727,181,412 | 1.11E-07 | 11.50 |
| E2 | AX-109428070 | 2A | 727,182,816 | 1.43E-06 | 11.51 |
| E2 | AX-109292249 | 2A | 727,183,443 | 1.11E-07 | 11.50 |
| E2 | AX-111509211 | 2A | 727,183,498 | 1.11E-07 | 11.50 |
| E2 | AX-108980791 | 2A | 727,187,444 | 1.11E-07 | 11.50 |
| E2 | AX-109961153 | 2A | 727,187,627 | 3.59E-08 | 14.31 |
| E2 | AX-108923021 | 2A | 727,190,555 | 5.39E-06 | 8.21 |
| E2 | AX-109373105 | 2A | 727,192,289 | 5.39E-06 | 8.21 |
| E2 | AX-111512779 | 2A | 727,192,510 | 8.65E-06 | 9.00 |
| E2 | AX-111502719 | 2A | 727,192,902 | 5.39E-06 | 8.21 |
| E2 | AX-109529880 | 2A | 727,195,932 | 5.39E-06 | 8.21 |
| E2 | AX-110447468 | 2A | 727,198,143 | 2.37E-06 | 8.52 |
| E2 | AX-108980644 | 2A | 727,243,309 | 3.07E-06 | 10.85 |
| E2 | AX-110362101 | 2A | 727,243,960 | 5.39E-06 | 8.21 |
| E2 | AX-109360792 | 2A | 727,245,925 | 5.30E-07 | 10.33 |
| E2 | AX-110397450 | 2A | 727,285,210 | 5.39E-06 | 8.21 |
| E2 | AX-108838121 | 2A | 727,614,295 | 6.78E-06 | 7.95 |
| E2 | AX-108908177 | 2A | 727,639,917 | 7.31E-06 | 7.71 |
| E2 | AX-109430725 | 2A | 727,658,142 | 7.31E-06 | 7.71 |
| E2 | AX-109290174 | 2A | 727,955,888 | 1.42E-06 | 10.99 |
| E2 | AX-111727771 | 2A | 728,020,803 | 1.72E-06 | 10.99 |
| E2 | AX-109884043 | 2A | 728,026,513 | 1.72E-06 | 10.99 |
| E2 | AX-108928895 | 2A | 728,029,569 | 3.16E-06 | 8.43 |
| E2 | AX-111628949 | 2A | 728,070,767 | 3.75E-06 | 8.30 |
| E2 | AX-111044883 | 2A | 728,072,564 | 3.30E-06 | 8.35 |
| E2 | AX-110624913 | 2A | 728,073,136 | 3.75E-06 | 8.30 |
| E2 | AX-109973650 | 2A | 728,132,521 | 1.72E-06 | 10.99 |
| E2 | AX-111505110 | 7A | 37,112,322 | 1.51E-05 | 4.46 |
| E2 | AX-86163556 | 7B | 610,735,918 | 9.93E-06 | 9.98 |
| E2 | AX-111609105 | 7B | 611,944,380 | 1.76E-05 | 7.33 |
| E2 | AX-111031595 | 7B | 611,960,672 | 1.72E-05 | 7.34 |
| B | AX-111651652 | 2A | 726,761,412 | 5.32E-06 | 11.91 |
| B | AX-108892693 | 2A | 726,762,422 | 7.42E-06 | 12.04 |
| B | AX-109972911 | 2A | 726,763,641 | 6.32E-07 | 11.44 |
| B | AX-109532923 | 2A | 726,763,898 | 6.32E-07 | 11.44 |
| B | AX-109449153 | 2A | 726,764,021 | 8.75E-07 | 11.48 |
| B | AX-111515528 | 2A | 726,770,069 | 9.62E-08 | 12.34 |
| B | AX-109323179 | 2A | 726,771,093 | 4.23E-09 | 13.49 |
| B | AX-110967398 | 2A | 726,772,232 | 1.40E-08 | 13.19 |
| B | AX-111698397 | 2A | 726,775,869 | 1.40E-08 | 13.19 |
| B | AX-111603918 | 2A | 726,776,128 | 4.82E-09 | 15.94 |
| B | AX-108793615 | 2A | 726,865,730 | 1.64E-08 | 13.19 |
| B | AX-108777425 | 2A | 726,971,223 | 1.40E-08 | 13.19 |
| B | AX-109930704 | 2A | 726,988,053 | 4.34E-09 | 15.14 |
| B | AX-110554284 | 2A | 726,994,887 | 2.11E-08 | 13.45 |
| B | AX-110492897 | 2A | 727,004,090 | 1.40E-08 | 13.19 |
| B | AX-110471201 | 2A | 727,005,198 | 1.40E-08 | 13.19 |
| B | AX-109390432 | 2A | 727,086,497 | 1.26E-05 | 9.54 |
| B | AX-110130904 | 2A | 727,141,584 | 1.40E-08 | 13.19 |
| B | AX-109884666 | 2A | 727,169,221 | 4.24E-07 | 12.77 |
| B | AX-109493384 | 2A | 727,170,336 | 7.46E-08 | 14.13 |
| B | AX-108792390 | 2A | 727,180,360 | 3.77E-09 | 15.94 |
| B | AX-111160521 | 2A | 727,181,412 | 1.63E-08 | 12.77 |
| B | AX-111079945 | 2A | 727,182,531 | 1.02E-05 | 8.63 |
| B | AX-109428070 | 2A | 727,182,816 | 1.45E-07 | 12.79 |
| B | AX-109292249 | 2A | 727,183,443 | 1.63E-08 | 12.77 |
| B | AX-111509211 | 2A | 727,183,498 | 1.63E-08 | 12.77 |
| B | AX-109415589 | 2A | 727,183,765 | 1.02E-05 | 8.63 |
| B | AX-108980791 | 2A | 727,187,444 | 1.63E-08 | 12.77 |
| B | AX-109961153 | 2A | 727,187,627 | 4.50E-09 | 15.48 |
| B | AX-109377277 | 2A | 727,189,359 | 4.63E-06 | 11.25 |
| B | AX-108923021 | 2A | 727,190,555 | 8.66E-07 | 9.55 |
| B | AX-109373105 | 2A | 727,192,289 | 8.66E-07 | 9.55 |
| B | AX-111512779 | 2A | 727,192,510 | 1.54E-06 | 10.15 |
| B | AX-111502719 | 2A | 727,192,902 | 8.66E-07 | 9.55 |
| B | AX-109529880 | 2A | 727,195,932 | 8.66E-07 | 9.55 |
| B | AX-110447468 | 2A | 727,198,143 | 3.52E-07 | 9.95 |
| B | AX-108817000 | 2A | 727,239,616 | 1.02E-05 | 8.63 |
| B | AX-108980644 | 2A | 727,243,309 | 6.81E-07 | 12.30 |
| B | AX-110362101 | 2A | 727,243,960 | 8.66E-07 | 9.55 |
| B | AX-109360792 | 2A | 727,245,925 | 3.43E-08 | 11.79 |
| B | AX-110397450 | 2A | 727,285,210 | 8.66E-07 | 9.55 |
| B | AX-109517909 | 2A | 727,388,731 | 1.02E-05 | 8.63 |
| B | AX-108838121 | 2A | 727,614,295 | 1.61E-06 | 9.59 |
| B | AX-109403176 | 2A | 727,627,448 | 1.35E-05 | 8.07 |
| B | AX-89631736 | 2A | 727,637,588 | 1.35E-05 | 8.07 |
| B | AX-108908177 | 2A | 727,639,917 | 1.25E-06 | 8.99 |
| B | AX-108809334 | 2A | 727,641,855 | 1.20E-05 | 8.08 |
| B | AX-111669509 | 2A | 727,655,211 | 1.35E-05 | 8.07 |
| B | AX-109430725 | 2A | 727,658,142 | 1.25E-06 | 8.99 |
| B | AX-108827577 | 2A | 727,951,545 | 1.24E-05 | 8.42 |
| B | AX-109290174 | 2A | 727,955,888 | 3.64E-07 | 11.94 |
| B | AX-108800266 | 2A | 728,018,251 | 1.24E-05 | 8.42 |
| B | AX-111064107 | 2A | 728,020,754 | 8.88E-06 | 9.45 |
| B | AX-111727771 | 2A | 728,020,803 | 4.51E-07 | 11.94 |
| B | AX-109884043 | 2A | 728,026,513 | 4.51E-07 | 11.94 |
| B | AX-108928895 | 2A | 728,029,569 | 1.29E-06 | 9.42 |
| B | AX-111628949 | 2A | 728,070,767 | 1.08E-06 | 9.34 |
| B | AX-111044883 | 2A | 728,072,564 | 9.58E-07 | 9.35 |
| B | AX-110624913 | 2A | 728,073,136 | 1.08E-06 | 9.34 |
| B | AX-111155137 | 2A | 728,073,306 | 1.24E-05 | 8.42 |
| B | AX-109973650 | 2A | 728,132,521 | 4.51E-07 | 11.94 |
| B | AX-109991080 | 2A | 728,132,822 | 1.24E-05 | 8.42 |
| B | AX-89664170 | 2A | 728,397,579 | 5.74E-06 | 11.04 |
| B | AX-110575901 | 2A | 728,403,416 | 7.74E-06 | 8.60 |
| B | AX-94481508 | 2A | 728,409,804 | 1.49E-05 | 8.32 |
| B | AX-109493253 | 2A | 728,409,806 | 1.24E-05 | 8.42 |
| B | AX-111609105 | 7B | 611,944,380 | 1.06E-05 | 5.76 |
| B | AX-111078922 | 7B | 611,956,721 | 1.72E-05 | 5.76 |
| B | AX-111031595 | 7B | 611,960,672 | 1.09E-05 | 5.81 |
| B | AX-109440948 | 7D | 611,243,842 | 1.99E-05 | 12.17 |
| B | AX-94482751 | 7D | 611,586,124 | 2.55E-05 | 13.88 |
| *E1: 2019, E2: 2020, B: BLUP of two years, PVE: phenotypic variance explained.* | | | | |  |

**Supplementary Table 3.** Genotypes of the three most significant SNPs in 207 wheat accessions.

| **Accession ID** | **AX-108792390** | **AX-111609105** | **AX-109440948** |
| --- | --- | --- | --- |
| W1 | TT | AA | GG |
| W2 | CC | AA | GG |
| W3 | TT | AA | GG |
| W4 | TT | AA | GG |
| W5 | CC | AA | GG |
| W6 | TT | AA | GG |
| W7 | TT | AA | GG |
| W8 | TT | AA | GG |
| W9 | TT | AA | GG |
| W10 | CC | GG | AA |
| W11 | CC | GG | GA |
| W12 | TT | AA | GG |
| W13 | TT | AA | GG |
| W14 | CC | AA | GA |
| W15 | TT | AA | GA |
| W16 | CC | GG | AA |
| W17 | CC | AA | GG |
| W18 | TT | AA | GG |
| W19 | TT | AA | GG |
| W20 | CC | AA | GG |
| W21 | CC | AA | GG |
| W22 | TT | AA | AA |
| W23 | TT | AA | GG |
| W24 | TT | AA | GG |
| W25 | TT | AA | GG |
| W26 | TT | AA | GG |
| W27 | TT | AA | AA |
| W28 | TT | AA | GG |
| W29 | TT | AA | GG |
| W30 | TT | AA | GG |
| W31 | TT | AA | GG |
| W32 | TT | AA | GG |
| W33 | TT | AA | GG |
| W34 | TT | AA | GG |
| W35 | TT | AA | GG |
| W36 | TT | AA | GG |
| W37 | TT | AA | GG |
| W38 | TT | AA | GG |
| W39 | TT | AA | GG |
| W40 | CC | GG | GG |
| W41 | TT | AA | GA |
| W42 | TT | AA | GA |
| W43 | TT | AA | GG |
| W44 | TT | AA | GG |
| W45 | TT | AA | GG |
| W46 | TT | AA | GG |
| W47 | CC | AA | GG |
| W48 | TT | AA | GG |
| W49 | TT | AA | GG |
| W50 | TT | AA | GG |
| W51 | CC | AA | GA |
| W52 | TT | AA | GG |
| W53 | CC | AA | GG |
| W54 | CC | AA | GA |
| W55 | CC | AA | GG |
| W56 | TT | AA | GA |
| W57 | TT | AA | GG |
| W58 | CC | AA | AA |
| W59 | TT | AA | GG |
| W60 | TT | AA | GG |
| W61 | TT | AA | GG |
| W62 | TT | AA | GG |
| W63 | TT | AA | GG |
| W64 | TT | AA | GG |
| W65 | TT | AA | GG |
| W66 | CC | AA | GG |
| W67 | TT | AA | GG |
| W68 | CC | AA | GG |
| W69 | CC | AA | GG |
| W70 | TT | AA | NN |
| W71 | CC | AA | GG |
| W72 | TT | AA | GG |
| W73 | TT | AA | GG |
| W74 | CC | AA | GG |
| W75 | TT | AA | GG |
| W76 | TT | AA | GG |
| W77 | TT | AA | GG |
| W78 | TT | AA | GG |
| W79 | CC | AA | GG |
| W80 | TT | AA | GG |
| W81 | CC | AA | GG |
| W82 | CC | AA | GA |
| W83 | CC | AA | GA |
| W84 | TT | AA | GG |
| W85 | TT | AA | GG |
| W86 | CC | AA | AA |
| W87 | TT | AA | GG |
| W88 | CC | AA | GG |
| W89 | CC | GG | AA |
| W90 | TT | AA | GG |
| W91 | CC | AA | GG |
| W92 | TT | AA | GG |
| W93 | TT | AA | GG |
| W94 | TT | AA | GG |
| W95 | CC | AA | GG |
| W96 | TT | AA | GA |
| W97 | TT | AA | GG |
| W98 | CC | AA | GG |
| W99 | TT | AA | GA |
| W100 | TT | AA | GA |
| W101 | TT | AA | GG |
| W102 | CC | AA | GG |
| W103 | CC | AA | GG |
| W104 | TT | AA | GG |
| W105 | TT | AA | GG |
| W106 | TT | AA | GG |
| W107 | TT | AA | GG |
| W108 | NN | AA | GG |
| W109 | TT | AA | GG |
| W110 | TT | AA | GG |
| W111 | TT | AA | GG |
| W112 | TT | AA | GG |
| W113 | TT | AA | GG |
| W114 | TT | AA | GA |
| W115 | TT | AA | GG |
| W116 | TT | AA | GG |
| W117 | TT | AA | GG |
| W118 | TT | AA | GG |
| W119 | TT | AA | GG |
| W120 | TT | AA | GA |
| W121 | TT | AA | GA |
| W122 | CC | GG | GG |
| W123 | TT | AA | GG |
| W124 | TT | AA | GG |
| W125 | TT | AA | GG |
| W126 | CC | GG | GG |
| W127 | TT | AA | GG |
| W128 | TT | AA | GG |
| W129 | CC | AA | AA |
| W130 | TT | AA | GG |
| W131 | CC | GG | AA |
| W132 | CC | AA | GG |
| W133 | TT | AA | GG |
| W134 | TT | AA | GG |
| W135 | TT | AA | GG |
| W136 | TT | AA | GG |
| W137 | TT | AA | GG |
| W138 | TT | AA | GG |
| W139 | TT | AA | GG |
| W140 | CC | AA | GA |
| W141 | TT | AA | GG |
| W142 | CC | AA | GG |
| W143 | TT | AA | GG |
| W144 | TT | AA | GG |
| W145 | CC | AA | GG |
| W146 | CC | GG | GG |
| W147 | CC | AA | GA |
| W148 | TT | AA | GG |
| W149 | CC | AA | GG |
| W150 | TT | AA | GG |
| W151 | TT | AA | GG |
| W152 | TT | GG | GG |
| W153 | CC | AA | GG |
| W154 | CC | AA | GG |
| W155 | TT | AA | GG |
| W156 | CC | AA | NN |
| W157 | CC | AA | GG |
| W158 | CC | GG | GG |
| W159 | CC | AA | GG |
| W160 | CC | AA | GG |
| W161 | TT | AA | GG |
| W162 | TT | AA | GG |
| W163 | TT | AA | GG |
| W164 | CC | AA | GA |
| W165 | TT | AG | GA |
| W166 | TT | GG | GG |
| W167 | CC | AA | GA |
| W168 | TT | AA | GA |
| W169 | TT | NN | GG |
| W170 | TT | AA | GG |
| W171 | TT | AA | GG |
| W172 | TT | AA | GG |
| W173 | CC | AA | GG |
| W174 | TT | AA | GG |
| W175 | TT | AA | GG |
| W176 | TT | AA | GG |
| W177 | TT | AA | GA |
| W178 | CC | AA | GG |
| W179 | CC | AA | GG |
| W180 | CC | AA | GG |
| W181 | CC | AA | GG |
| W182 | CC | AA | GG |
| W183 | TT | AA | GG |
| W184 | TT | AA | GG |
| W185 | CC | AA | GG |
| W186 | TT | AA | GA |
| W187 | CC | AA | GG |
| W188 | CC | AA | GG |
| W189 | TT | AA | GG |
| W190 | CC | AA | GA |
| W191 | CC | GG | GG |
| W192 | TT | AA | GG |
| W193 | CC | GG | GG |
| W194 | CC | GG | GG |
| W195 | CC | GG | GG |
| W196 | CC | GG | GG |
| W197 | CC | GG | GG |
| W198 | CC | AA | GG |
| W199 | CC | GG | GG |
| W200 | CC | AA | GG |
| W201 | CC | GG | GG |
| W202 | CC | AA | AA |
| W203 | TT | GG | AA |
| W204 | TT | AA | GA |
| W205 | CC | GG | AA |
| W206 | TT | AA | GA |
| W207 | CC | AA | AA |

**Supplementary Table 4.** Expression and closely associated SNP genotypes of the three candidate genes in 207 wheat accessions.

| **Accession ID** | **AX-109360792** | **AX-109290174** | **AX-111628949** | **AX-111044883** | **TraesCS2A02G496200 (FPKM)** | **TraesCS2A02G496700 (FPKM)** | **TraesCS2A02G497200 (FPKM)** |
| --- | --- | --- | --- | --- | --- | --- | --- |
| W1 | GG | GG | GG | CC | 0.51 | 0.03 | 5.31 |
| W2 | AA | AA | AA | GG | 5.01 | 0.02 | 7.25 |
| W3 | GG | GG | GG | CC | 2.07 | 0.06 | 5.44 |
| W4 | GG | GG | GG | CC | 1.59 | 0.00 | 7.73 |
| W5 | AA | AA | AA | GG | 1.96 | 0.00 | 4.35 |
| W6 | GG | GG | GG | CC | 3.96 | 0.00 | 6.06 |
| W7 | GG | GG | GG | CC | 0.21 | 0.00 | 5.70 |
| W8 | GG | GG | GG | CC | 0.68 | 0.00 | 8.60 |
| W9 | GG | GG | GG | CC | 3.00 | 0.01 | 9.73 |
| W10 | AA | AA | AA | GG | 2.01 | 0.03 | 7.31 |
| W11 | AA | AA | AA | GG | 1.63 | 0.00 | 6.79 |
| W12 | GG | GG | GG | CC | 0.33 | 0.00 | 7.54 |
| W13 | GG | GG | GG | CC | 0.49 | 0.05 | 7.62 |
| W14 | AA | AA | AA | GG | 1.30 | 0.17 | 5.93 |
| W15 | GG | GG | GG | CC | 0.94 | 0.25 | 3.82 |
| W16 | AA | AA | AA | GG | 0.61 | 0.00 | 5.68 |
| W17 | AA | AA | AA | GG | 2.33 | 0.00 | 5.65 |
| W18 | GG | GG | GG | CC | 2.95 | 0.00 | 6.67 |
| W19 | GG | GG | GG | CC | 0.32 | 0.00 | 6.48 |
| W20 | AA | AA | AA | GG | 3.51 | 0.10 | 6.80 |
| W21 | AA | AA | AA | GG | 2.64 | 0.17 | 5.39 |
| W22 | GG | GG | GG | CC | 0.48 | 0.01 | 8.84 |
| W23 | GG | GG | GG | CC | 2.48 | 0.04 | 13.70 |
| W24 | AA | GG | GG | CC | 1.21 | 0.22 | 8.32 |
| W25 | GG | GG | GG | CC | 0.23 | 0.30 | 11.63 |
| W26 | GG | GG | GG | CC | 0.35 | 0.03 | 9.21 |
| W27 | GG | GG | GG | CC | 0.35 | 0.04 | 8.28 |
| W28 | GG | GG | GG | CC | 1.36 | 0.00 | 12.60 |
| W29 | GG | GG | GG | CC | 0.29 | 0.00 | 6.82 |
| W30 | GG | GG | GG | CC | 0.25 | 0.00 | 7.58 |
| W31 | GG | GG | GG | CC | 0.22 | 0.07 | 11.95 |
| W32 | GG | GG | GG | CC | 0.21 | 0.25 | 10.74 |
| W33 | GG | GG | GG | CC | 0.52 | 0.00 | 11.10 |
| W34 | GG | GG | GG | CC | 2.76 | 0.30 | 8.05 |
| W35 | GG | GG | GG | CC | 0.24 | 0.22 | 6.35 |
| W36 | GG | GG | GG | CC | 1.29 | 0.03 | 11.14 |
| W37 | GG | GG | GG | CC | 0.15 | 0.00 | 9.89 |
| W38 | GG | GG | GG | CC | 0.85 | 0.00 | 9.21 |
| W39 | GG | GG | GG | CC | 0.61 | 0.09 | 6.51 |
| W40 | GG | GG | GG | CC | 0.64 | 0.00 | 8.66 |
| W41 | GG | GG | GG | CC | 0.44 | 0.06 | 12.56 |
| W42 | GG | GG | GG | CC | 1.07 | 0.02 | 9.00 |
| W43 | GG | GG | GG | CC | 0.57 | 0.00 | 11.88 |
| W44 | GG | GG | GG | CC | 0.79 | 0.00 | 6.59 |
| W45 | GG | GG | GG | CC | 0.58 | 0.00 | 10.26 |
| W46 | GG | GG | GG | CC | 0.99 | 0.00 | 7.70 |
| W47 | AA | AA | AA | GG | 4.27 | 0.00 | 6.13 |
| W48 | GG | GG | GG | CC | 0.16 | 0.00 | 8.36 |
| W49 | GG | GG | GG | CC | 0.45 | 0.00 | 9.11 |
| W50 | GG | GG | GG | CC | 4.57 | 0.02 | 9.97 |
| W51 | AA | AA | AA | GG | 1.94 | 0.00 | 7.25 |
| W52 | GG | GG | GG | CC | 0.54 | 0.03 | 6.59 |
| W53 | AA | AA | AA | GG | 2.16 | 0.00 | 7.87 |
| W54 | AA | AA | AA | GG | 1.40 | 0.00 | 5.20 |
| W55 | AA | AA | AA | GG | 2.38 | 0.00 | 5.58 |
| W56 | GG | GG | GG | CC | 1.57 | 0.11 | 6.75 |
| W57 | GG | GG | GG | CC | 0.37 | 0.00 | 5.72 |
| W58 | AA | AA | AA | GG | 2.06 | 0.01 | 7.20 |
| W59 | GG | GG | GG | CC | 0.09 | 0.05 | 9.67 |
| W60 | GG | GG | GG | CC | 0.69 | 0.04 | 8.21 |
| W61 | GG | GG | GG | CC | 0.61 | 0.09 | 11.79 |
| W62 | GG | GG | GG | CC | 4.38 | 0.03 | 7.18 |
| W63 | GG | GG | GG | CC | 0.56 | 0.01 | 7.94 |
| W64 | GG | GG | GG | CC | 0.35 | 0.33 | 7.04 |
| W65 | GG | GG | GG | CC | 0.90 | 0.00 | 5.74 |
| W66 | AA | AA | AA | GG | 6.08 | 0.18 | 6.71 |
| W67 | GG | GG | GG | CC | 5.89 | 0.00 | 6.99 |
| W68 | AA | AA | AA | GG | 8.10 | 0.10 | 5.53 |
| W69 | AA | AA | AA | GG | 2.23 | 0.00 | 4.49 |
| W70 | GG | GG | GG | CC | 3.73 | 0.00 | 5.35 |
| W71 | AA | AA | AA | GG | 5.30 | 0.00 | 5.58 |
| W72 | GG | GG | GG | CC | 1.98 | 0.00 | 9.45 |
| W73 | GG | GG | GG | CC | 1.92 | 0.03 | 7.30 |
| W74 | AA | AA | AA | GG | 2.74 | 0.00 | 6.96 |
| W75 | GG | GG | GG | CC | 0.97 | 0.08 | 5.35 |
| W76 | GG | GG | GG | CC | 1.57 | 0.00 | 7.87 |
| W77 | GG | GG | GG | CC | 2.94 | 0.00 | 6.26 |
| W78 | GG | GG | GG | CC | 4.82 | 0.00 | 8.65 |
| W79 | AA | AA | AA | GG | 3.00 | 0.00 | 8.11 |
| W80 | GG | GG | GG | CC | 1.37 | 0.08 | 5.55 |
| W81 | AA | AA | AA | GG | 1.60 | 0.09 | 5.22 |
| W82 | AA | AA | AA | GG | 3.17 | 0.00 | 6.74 |
| W83 | AA | AA | AA | GG | 6.90 | 0.10 | 7.15 |
| W84 | GG | GG | GG | CC | 0.98 | 0.06 | 7.72 |
| W85 | GG | GG | GG | CC | 1.20 | 0.03 | 5.96 |
| W86 | AA | AA | AA | GG | 1.62 | 0.00 | 5.16 |
| W87 | GG | GG | GG | CC | 0.40 | 0.09 | 8.43 |
| W88 | AA | AA | AA | GG | 2.55 | 0.09 | 11.13 |
| W89 | AA | AA | AA | GG | 1.89 | 0.00 | 8.85 |
| W90 | GG | GG | GG | CC | 0.33 | 0.04 | 11.00 |
| W91 | AA | AA | AA | GG | 2.62 | 0.01 | 7.23 |
| W92 | GG | GG | GG | CC | 1.76 | 0.14 | 9.79 |
| W93 | GG | GG | GG | CC | 0.40 | 0.07 | 7.58 |
| W94 | GG | GG | GG | CC | 0.69 | 0.04 | 6.48 |
| W95 | AA | AA | AA | GG | 3.42 | 0.08 | 9.39 |
| W96 | GG | GG | GG | CC | 0.35 | 0.01 | 8.10 |
| W97 | GG | GG | GG | CC | 0.17 | 0.00 | 11.66 |
| W98 | AA | AA | AA | GG | 5.33 | 0.00 | 9.09 |
| W99 | GG | GG | GG | CC | 5.58 | 0.05 | 7.45 |
| W100 | GG | GG | GG | CC | 0.30 | 0.02 | 8.70 |
| W101 | GG | GG | GG | CC | 3.13 | 0.00 | 6.48 |
| W102 | AA | AA | AA | GG | 2.99 | 0.00 | 8.48 |
| W103 | AA | AA | AA | GG | 1.33 | 0.04 | 5.04 |
| W104 | GG | GG | GG | CC | 3.49 | 0.02 | 8.80 |
| W105 | GG | GG | GG | CC | 0.74 | 0.07 | 8.61 |
| W106 | GG | GG | GG | CC | 0.66 | 0.00 | 5.35 |
| W107 | GG | GG | GG | CC | 0.71 | 0.09 | 11.42 |
| W108 | NN | NN | AA | GG | 2.67 | 0.00 | 9.26 |
| W109 | GG | GG | GG | CC | 0.16 | 0.00 | 8.04 |
| W110 | GG | GG | GG | CC | 1.49 | 0.90 | 8.80 |
| W111 | GG | GG | GG | CC | 2.35 | 0.02 | 5.54 |
| W112 | GG | GG | GG | CC | 2.70 | 0.02 | 10.27 |
| W113 | GG | GG | GG | CC | 5.54 | 0.00 | 11.32 |
| W114 | GG | GG | GG | CC | 0.30 | 0.09 | 8.06 |
| W115 | GG | GG | GG | CC | 1.20 | 0.29 | 10.20 |
| W116 | GG | GG | GG | CC | 0.59 | 0.03 | 9.44 |
| W117 | GG | GG | GG | CC | 3.12 | 0.65 | 12.97 |
| W118 | GG | GG | GG | CC | 0.84 | 0.01 | 8.26 |
| W119 | GG | GG | GG | CC | 0.23 | 0.08 | 6.71 |
| W120 | NN | GG | GG | CC | 3.90 | 0.00 | 8.70 |
| W121 | NN | GG | GG | CC | 6.98 | 0.00 | 9.74 |
| W122 | AA | AA | AA | GG | 2.96 | 0.01 | 9.99 |
| W123 | GG | GG | GG | CC | 1.14 | 0.06 | 12.18 |
| W124 | GG | GG | GG | CC | 0.21 | 0.00 | 9.21 |
| W125 | GG | GG | GG | CC | 0.90 | 0.09 | 10.13 |
| W126 | AA | AA | AA | GG | 1.45 | 0.00 | 8.12 |
| W127 | GG | GG | GG | CC | 0.90 | 0.00 | 9.94 |
| W128 | GG | GG | GG | CC | 2.90 | 0.00 | 7.90 |
| W129 | AA | AA | AA | GG | 1.69 | 0.00 | 4.81 |
| W130 | GG | GG | GG | CC | 0.14 | 0.04 | 9.14 |
| W131 | AA | AA | AA | GG | 1.41 | 0.00 | 8.25 |
| W132 | AA | AA | AA | GG | 3.32 | 0.00 | 6.24 |
| W133 | GG | GG | GG | CC | 1.47 | 0.10 | 6.65 |
| W134 | GG | GG | GG | CC | 0.40 | 0.07 | 6.62 |
| W135 | GG | GG | GG | CC | 0.24 | 0.04 | 7.78 |
| W136 | GG | GG | GG | CC | 1.16 | 0.02 | 6.31 |
| W137 | GG | GG | GG | CC | 4.08 | 0.00 | 7.94 |
| W138 | GG | GG | GG | CC | 4.21 | 0.01 | 7.57 |
| W139 | GG | GG | GG | CC | 4.02 | 0.00 | 11.20 |
| W140 | AA | AA | AA | GG | 2.37 | 0.10 | 6.63 |
| W141 | GG | GG | GG | CC | 5.12 | 0.02 | 11.98 |
| W142 | AA | AA | AA | GG | 1.60 | 0.00 | 9.22 |
| W143 | GG | GG | GG | CC | 0.37 | 0.03 | 6.03 |
| W144 | GG | GG | GG | CC | 0.50 | 0.03 | 8.50 |
| W145 | AA | AA | AA | GG | 3.79 | 0.02 | 4.40 |
| W146 | AA | AA | AA | GG | 5.79 | 0.15 | 7.53 |
| W147 | AA | AA | AA | GG | 6.45 | 0.06 | 9.00 |
| W148 | GG | GG | GG | CC | 1.08 | 0.18 | 4.55 |
| W149 | AA | AA | AA | GG | 1.79 | 0.11 | 5.36 |
| W150 | GG | GG | GG | CC | 2.32 | 0.13 | 5.47 |
| W151 | GG | GG | GG | CC | 0.24 | 0.00 | 6.20 |
| W152 | GG | GG | GG | CC | 3.14 | 0.00 | 7.92 |
| W153 | AA | AA | AA | GG | 1.82 | 0.01 | 5.68 |
| W154 | AA | AA | AA | GG | 2.15 | 0.02 | 6.67 |
| W155 | GG | GG | GG | CC | 1.62 | 0.10 | 7.53 |
| W156 | AA | AA | AA | GG | 1.15 | 0.00 | 5.65 |
| W157 | AA | AA | AA | GG | 5.19 | 0.12 | 6.37 |
| W158 | AA | AA | AA | GG | 1.84 | 0.00 | 7.15 |
| W159 | AA | AA | AA | GG | 0.68 | 0.00 | 8.94 |
| W160 | AA | AA | AA | GG | 3.50 | 0.06 | 7.82 |
| W161 | GG | GG | GG | CC | 4.99 | 0.00 | 10.52 |
| W162 | GG | GG | GG | CC | 0.79 | 0.08 | 8.34 |
| W163 | GG | GG | GG | CC | 0.48 | 0.23 | 10.32 |
| W164 | GG | GG | GG | CC | 0.79 | 0.00 | 15.08 |
| W165 | GG | GG | GG | CC | 0.85 | 0.03 | 6.94 |
| W166 | GG | GG | GG | CC | 0.39 | 0.00 | 9.16 |
| W167 | GG | GG | GG | CC | 0.28 | 0.00 | 9.70 |
| W168 | GG | GG | GG | CC | 0.72 | 0.01 | 12.13 |
| W169 | GG | GG | GG | NN | 0.36 | 0.00 | 6.90 |
| W170 | GG | GG | GG | CC | 0.00 | 0.00 | 11.31 |
| W171 | GG | GG | GG | CC | 6.80 | 0.01 | 9.92 |
| W172 | GG | GG | GG | CC | 0.10 | 0.00 | 8.69 |
| W173 | AA | AA | AA | GG | 1.48 | 0.00 | 5.82 |
| W174 | GG | GG | GG | CC | 0.85 | 0.02 | 10.89 |
| W175 | GG | GG | GG | CC | 0.03 | 0.02 | 7.58 |
| W176 | GG | GG | GG | CC | 0.14 | 0.00 | 8.30 |
| W177 | GG | AA | AA | GG | 0.36 | 0.00 | 6.71 |
| W178 | AA | AA | AA | GG | 1.76 | 0.03 | 6.45 |
| W179 | AA | AA | AA | GG | 2.23 | 0.00 | 6.74 |
| W180 | AA | AA | AA | GG | 1.72 | 0.00 | 6.95 |
| W181 | AA | AA | AA | GG | 1.22 | 0.08 | 9.46 |
| W182 | AA | AA | AA | GG | 1.87 | 0.13 | 7.74 |
| W183 | GG | GG | GG | CC | 0.90 | 0.00 | 6.93 |
| W184 | GG | GG | GG | CC | 2.56 | 0.00 | 7.79 |
| W185 | AA | AA | AA | GG | 6.40 | 0.01 | 5.67 |
| W186 | GG | GG | GG | CC | 0.48 | 0.00 | 7.52 |
| W187 | AA | AA | AA | GG | 2.73 | 0.00 | 5.24 |
| W188 | AA | AA | AA | GG | 3.59 | 0.00 | 6.81 |
| W189 | GG | GG | GG | CC | 1.46 | 0.09 | 6.65 |
| W190 | AA | AA | AA | GG | 0.10 | 0.07 | 7.74 |
| W191 | AA | AA | AA | GG | 1.05 | 0.08 | 6.89 |
| W192 | GG | GG | GG | CC | 0.57 | 0.04 | 8.08 |
| W193 | AA | AA | AA | GG | 3.25 | 0.01 | 9.53 |
| W194 | AA | AA | AA | GG | 6.03 | 0.03 | 7.09 |
| W195 | AA | AA | AA | GG | 1.46 | 0.03 | 6.24 |
| W196 | AA | AA | AA | GG | 2.26 | 0.00 | 10.40 |
| W197 | AA | AA | AA | GG | 1.22 | 0.03 | 7.50 |
| W198 | AA | AA | AA | GG | 2.97 | 0.11 | 7.73 |
| W199 | AA | AA | AA | GG | 4.11 | 0.19 | 7.57 |
| W200 | AA | AA | AA | GG | 2.45 | 0.17 | 7.59 |
| W201 | AA | AA | AA | GG | 3.17 | 0.09 | 6.29 |
| W202 | AA | AA | AA | GG | 3.28 | 0.00 | 9.24 |
| W203 | NN | GG | GG | CC | 2.26 | 0.00 | 7.54 |
| W204 | GG | GG | GG | CC | 0.33 | 0.00 | 8.13 |
| W205 | AA | AA | AA | GG | 2.59 | 0.00 | 6.87 |
| W206 | NN | GG | GG | CC | 4.11 | 0.06 | 13.24 |
| W207 | AA | AA | AA | GG | 0.83 | 0.03 | 3.54 |
